# Study Protocol: Longitudinal Attention and Temperament Study

**DOI:** 10.3389/fpsyt.2021.656958

**Published:** 2021-06-08

**Authors:** Koraly Pérez-Edgar, Vanessa LoBue, Kristin A. Buss, Andy P. Field, The LAnTs Team, Lori Reider

**Affiliations:** ^1^Department of Psychology, The Pennsylvania State University, University Park, PA, United States; ^2^Department of Psychology, Rutgers University, Newark, NJ, United States; ^3^School of Psychology, University of Sussex, Brighton, United Kingdom

**Keywords:** attention, temperament, anxiety, eye-tracking, EEG, longitudinal, infancy

## Abstract

**Background:** Attention processes may play a central role in shaping trajectories of socioemotional development. Individuals who are clinically anxious or have high levels of trait anxiety sometimes show attention biases to threat. There is emerging evidence that young children also demonstrate a link between attention bias to salient stimuli and broad socioemotional profiles. However, we do not have a systematic and comprehensive assessment of how attention biases, and associated neural and behavioral correlates, emerge and change from infancy through toddlerhood. This paper describes the Longitudinal Attention and Temperament study (LAnTs), which is designed to target these open questions.

**Method:** The current study examines core components of attention across the first 2 years of life, as well as measures of temperament, parental psychosocial functioning, and biological markers of emotion regulation and anxiety risk. The demographically diverse sample (*N* = 357) was recruited from the area surrounding State College, PA, Harrisburg, PA, and Newark, NJ. Infants and parents are assessed at 4, 8, 12, 18, and 24 months. Assessments include repeated measures of attention bias (via eye-tracking) in both infants and parents, and measures of temperament (reactivity, negative affect), parental traits (e.g., anxiety and depression), biological markers (electrophysiology, EEG, and respiratory sinus arrythmia, RSA), and the environment (geocoding, neighborhood characteristics, perceived stress). Outcomes include temperamental behavioral inhibition, social behavior, early symptom profiles, and cellular aging (e.g., telomere length).

**Discussion:** This multi-method study aims to identify biomarkers and behavioral indicators of attentional and socioemotional trajectories. The current study brought together innovative measurement techniques to capture the earliest mechanisms that may be causally linked to a pervasive set of problem behaviors. The analyses the emerge from the study will address important questions of socioemotional development and help shape future research. Analyses systematically assessing attention bias patterns, as well as socioemotional profiles, will allow us to delineate the time course of any emerging interrelations. Finally, this study is the first to directly assess competing models of the role attention may play in socioemotional development in the first years of life.

## Background

The centrality of attention in development grows out of its role as a specific brain-based mechanism whose core function is to influence the operation of other mechanisms—by choosing the focus of attention for further processing, by maintaining this focus as needed, and by disengaging from the focus of attention when it no longer serves current goals (1). The earliest forms of self-regulation are rooted in the ability to disengage, shift gaze, and re-orient on a new focus of attention (2). In this way, attention mechanisms may play a pivotal role in shaping the individual's experienced environment from the first days of life (3). An emerging literature points to a potential causal association between attention (particularly attention bias to threat) and the presence of clinical and trait anxious behaviors in adults and children (4, 5). Attention bias refers to selective attention processes that preferentially select for and process specific categories of salient stimuli (6). There is some evidence that systematic biases toward and away from threat may play a causal or sustaining role in the emergence of disorder (7). In the anxiety literature, threat is often conveyed with the use of negative faces (e.g., angry or sad), particularly when examining social anxiety (7).

If this view is correct, individual differences in attention, first emerging in infancy, should be associated with diverging trajectories of socioemotional development. These trajectories may be potentiated among children at temperamental risk for anxiety (8) or children exposed to anxiogenic environments (9). In particular, the evident link between early temperamental negative affect and the later emergence of anxiety may be potentiated by the added presence of an attention bias to threat (10, 11). Although we are unable to follow the full emergence of anxiety in the first 2 years, we can capture the processes that may lay an initial developmental foundation. Understanding these early relations could thus provide avenues for (1) understanding mechanisms that lead to the emergence of social withdrawal and anxiety and (2) identifying individuals at risk for socioemotional difficulties. Taken together, this knowledge would help the field focus on specific windows of intervention, targeting causal mechanisms while the system is still plastic and malleable.

However, the literature to date cannot directly provide the needed data because it has focused on older children and adults when examining the relation between attention, affect, and socioemotional functioning. In addition, the data generated are from predominantly single-session, cross-sectional designs focusing on individuals already presenting with clear signs of clinical anxiety or trait-level distress (12, 13). Much of the developmentally-informed research on anxiety has focused on the classification and treatment of disorder (14). Although there is increasingly more data available with child samples, we have scant knowledge of normative or maladaptive developmental trajectories in infancy (15, 16). As such, it is not clear how attention patterns come to be associated with affect and how these constructs, together, underlie the emergence of anxiety.

**Figure 1:**
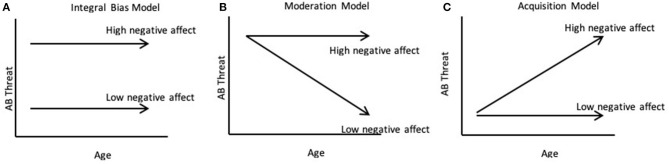
Schematic illustration of the three models for the emergence of attention bias in the first years of life based on Field and Lester (17). The integral bias, moderation, and acquisition models differ in the timing, stability, and sensitivity to outside factors for attention processes.

Field and Lester (17) suggested three potential developmental models of attention bias (Figure 1 illustrates the models using temperament as the potential developmental moderator). The integral bias model (18) suggests that the magnitude of any information processing is determined by individual factors (e.g., anxiety, temperament) and should be evident and fairly stable across the lifespan, assuming that it is measured using a developmentally appropriate task (Figure 1A). As such, infants with early signs of negative affect would already show a more pronounced bias to threat relative to infants without this temperamental profile. Much of the current clinical literature makes this *implicit* assumption. The moderation model (17) suggests that development moderates the expression of an existing bias to threat (Figure 1B), such that under certain circumstances (e.g., in children at temperamental risk for anxiety) the initial normative bias may be linked to the later emergence of elevated fear and social withdrawal (6, 19, 20). In contrast, normative biases will decrease over time for children with low temperamental risk. Finally, the acquisition model (Figure 1C) suggests that developmental experiences shape the acquisition of an attention bias gradually over time (17), either in tandem or subsequent to the emergence of fear and anxiety.

**Figure 2:**
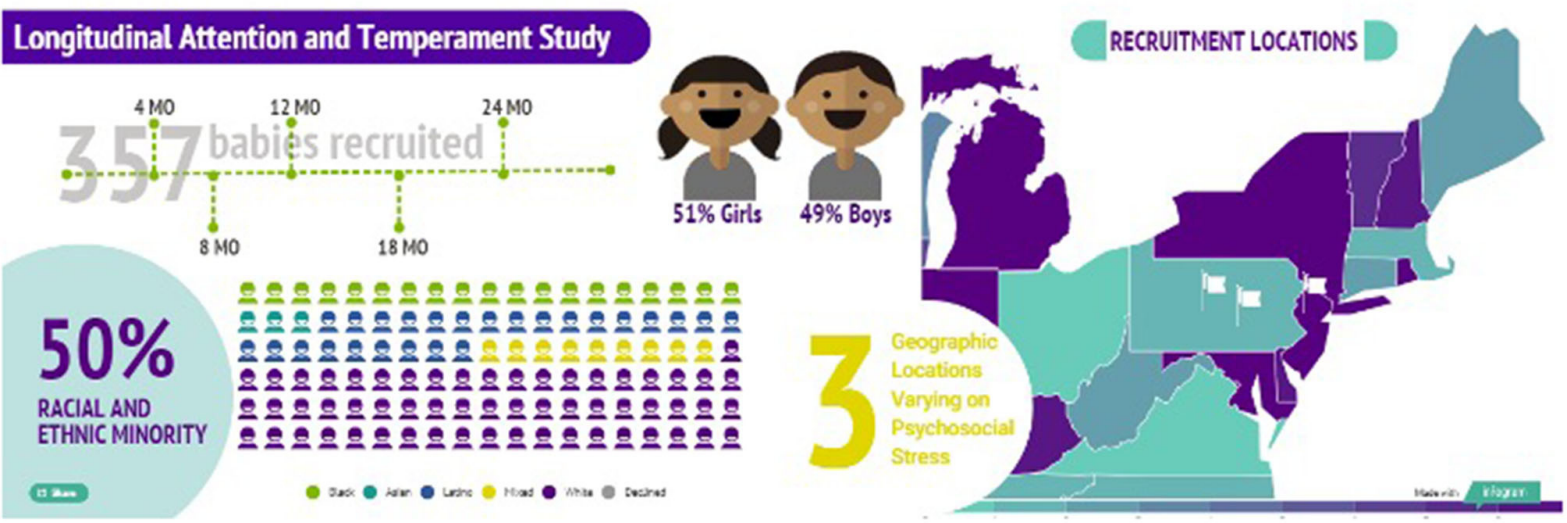
Schematic of the LAnTs cohort. Families are recruited from areas surrounding three locations, State College, PA, Harrisburg, PA, and Newark, NJ. The sample was recruited primarily at 4 months of age, and then assessed again at 8, 12, 18, and 24 months.

Testing these models, and examining the broader assumptions regarding attention-emotion relations, requires systematic studies that examine individual differences (21) across multiple levels of analysis (22) over time. Our prior work has examined associated questions in cross-sectional samples (23–27). The Longitudinal Attention and Temperament study (LAnTs; Figure 2) was designed to extend this work by bringing together three developmentally-appropriate tasks (dot-probe, overlap, vigilance) that can be used across the first 2 years of life (26). In addition, we assess early temperament using both observed behavior and parental reports. To identify endogenous factors that may modulate developmental risk, we assess resting electroencephalogram (EEG) to capture measures associated with emotion regulation and socioemotional risk, including frontal EEG alpha asymmetry (28), delta-beta coupling (29), and neural noise (30). We also capture respiratory sinus arrythmia (RSA) at rest and during our temperament battery (31) to examine peripheral markers of regulation (32). Given the central role parents play in shaping the daily experiences of children, contextual measures of parental attention bias, symptomatology, and psychosocial stress are assessed at every time point. Finally, we incorporate both objective (e.g., geocoding) and subjective (e.g., perceived violence and support) measures of the child's broader environment (33). Across levels of analysis the protocol generates a multidimensional profile of the individual and nested layers of development from the micro- through the mesosystem over time (34). LAnTs was designed to examine two core aims:

First, we will test the integral bias, moderation, and acquisition models outlined by Field and Lester (17). The first step will be to quantify the developmental trajectory (i.e., growth curve) of attention to threat. Each developmental model makes a unique prediction regarding how individual, biological, and environmental moderators will affect the size and direction of the developmental trajectory of attentional bias over time. Therefore, we will quantify the extent to which individual differences in negative affect moderate attention trajectories. We will then do the same analysis incorporating individual biomarkers (EEG and RSA). Finally, we will turn our focus on contextual factors (parental attention bias, symptomatology, psychosocial stress).

Second, we will examine the extent to which the gradient of individual attention growth curves predicts behavioral inhibition at age two. We will also capture potential behavioral, biological and contextual moderators of these individual gradients, particularly if the acquisition or moderation models are supported. As part of these outcome assessments, we will also examine early measures of psychopathology (35) and biological measures of chronic stress [e.g., telomere length (36)]. Greater detail regarding the larger analytic approach is provided in the Supplementary Materials.

The purpose of this paper is to provide a detailed description of the LAnTs protocol, measures, and sample. This will help place future analyses within the context of the full protocol. In addition, interested researchers may determine that the sample provides data needed for ancillary analyses.

## Methods

### General Procedure

We collected data from infants (*N* = 357) longitudinally at 4, 8, 12, 18, and 24 months, using a multi-method approach (see Table 1). At all five time-points the infant protocol included 3 eye-tracking tasks and a behavioral temperament battery [reactivity (37) at 4 months and the Laboratory Temperament Assessment Battery (Lab-TAB) (38) at 8–24 months]. At these visits, parents also completed two eye-tracking tasks and questionnaires assessing infant temperament, their own psychological state and traits, and the sociodemographic features of their environment. Geocoding was used as an additional measure of the familial environment.

**Table 1:** List of LAnTS measures by time point of data collection.

	**Description**	**4-mo**	**8-mo**	**12-mo**	**18-mo**	**24-mo**
**Eye-tracking measures**						
Baby dot-probe task (infant)	Attention bias task	X	X	X	X	X
Overlap task (infant)	Attention orienting	X	X	X	X	X
Vigilance task (infant)	Attention vigilance	X	X	X	X	X
Adult dot-probe task (parent)	Attention bias task	X	X	X	X	X
Adult vigilance task (parent)	Attention vigilance	X	X	X	X	X
**Behavioral measures**						
THISTLE reactivity coding	Infant reactivity to novelty	X				
Lab TAB	Infant temperament		X	X	X	X
Free play	Mother-child dyadic play		X	X	X	X
**Parental-report infant measures**						
Infant behavior questionnaire (IBQ-R)	Infant temperament	X	X	X		
Toddler behavior assessment questionnaire (TBAQ)	Toddler temperament			X	X	X
Infant-Toddler socioemotional assessment (ITSEA)	Toddler social-emotional problems			X	X	X
Child behavior checklist (CBCL)	Childhood anxiety				X	X
MacArthur-Bates communicative development inventory short form (MB-CDI-SF)	Child language development					X
**Parental personality and symptomatology**						
Adult temperament questionnaire (ATQ)	Parent temperament	X				
Eysenck personality questionnaire (EPQ)	Parent personality	X				
Check & buss shyness scale (CBSS)	Parent shyness	X				
Adult measure of behavioral inhibition (AMBI)	Parent behavioral inhibition	X				
Retrospective measure of behavioral inhibition (RMBI)	Parent behavioral inhibition	X				
Positive and negative affect scale (PANAS)	Parent emotionality	X	X	X	X	X
State-trait anxiety inventory (STAI)	Parent anxiety (trait)	X	X	X	X	X
Beck anxiety inventory (BAI)	Parent anxiety (state)	X	X	X	X	X
Beck depression inventory (BDI)	Parent depression	X	X	X	X	X
**Parental psychosocial stressors**						
ICPSR community survey	Environmental stress	X	X	X	X	X
Confusion, hubbub, and order scale (CHAOS)	Disorganization in the home	X	X	X	X	X
Parent daily hassle survey (PDHS-R)	Stressful life events	X	X	X	X	X
Geocoding	Environmental risk	X	X	X	X	X
**Biomarkers of risk**						
EEG at rest	EEG asymmetry & coherence		X	X	X	X
RSA during the lab TAB and dyad	Parasympathetic response		X	X	X	X
Telomere length assays	Aging and stress exposure					X
**Behavioral inhibition**						
Social dyad & individual protocol	Social behavior and novelty					X

*For families enrolled after 4 months, the ATQ, EPQ, CBSS, AMBI, and RMBI were recorded at the first visit*.

At the latter four time-points participants engaged in a structured parent-infant interaction. Infants also provided resting EEG and RSA. RSA data were also collected during the behavioral temperament battery and parent-child dyads. At 24 months, infants completed a behavioral inhibition (BI) protocol and engaged in an additional social dyad with an unfamiliar same-age peer. At this final visit, buccal swabs were collected from parents and infants for telomere length assays.

Data collection was generally completed in two, 2-h visits to the lab for the first four timepoints, although some families completed all tasks in a single visit, and a subset of families required three visits. During Visit 1, the infant and the primary caregiver typically completed the eye tracking tasks, with the infant first, followed by the caregiver. At 4 months, the eye-tracking and behavioral measures were usually all collected in a single day. For the 8- through 24-month time points, resting EEG was collected during Visit 2, followed by free play, and the Lab TAB episodes. RSA was collected throughout the behavioral tasks. The majority of visits followed this structure, but task orders sometimes varied based on the infant's needs. Most caregivers completed the online questionnaires at home prior to the visit, but in some cases, they were completed in the lab or over the phone. If questionnaires had to be completed in the lab, primary caregivers would do so while the infant was completing the eye tracking tasks or after data collection was completed. The social dyad was completed on a separate day, in a final visit to the lab at 24 months.

A detailed description of each measure (see also Table 1) can be found in the Supplemental Materials.

## Sample Characteristics

Here we highlight core metrics that describe and characterize the sample at the time of enrollment. Data collection is still ongoing through Fall 2021.

### Sample

Participants were recruited through local baby registries (40% families) and university-sponsored participant databases (13% families). In addition, we used a variety of community-level recruitment strategies, such as visiting local lactation/parenting classes, communicating with families at local community events, and talking to parents at local hospitals, health care centers, and Women's and Infant Centers (WIC). Community recruiting identified 38% of our families. The remaining 10% of families were recruited by word-of-mouth. Prospective families were contacted by letter, email, or phone explaining the motivations and methods of the study. The Institutional Review Boards at the Pennsylvania State University and Rutgers University approved all procedures and parents provided written consent and were compensated for their participation.

Infants and their caregivers were enrolled when the infants were 4 months of age (*N* = 298; 151 males, 147 females; *M*_age_ = 4.80 months; *SD*_age_ = 0.80, *Range*_*age*_ = 3.27–7.60 months), with an additional 46 participants enrolled at 8 months (*N* = 46; 19 males, 27 females; *M*_age_ = 8.83 months; *SD*_age_ = 0.73, *Range*_*age*_ = 7.53–10.20 months), and 13 participants at 12 months (*N* = 13; six males, seven females; *M*_age_ = 12.73 months; *SD*_age_ = 1.12, *Range*_*age*_ = 10.63–14.90 months), for a total enrollment of 357 infants in the full sample (176 males, 181 females). Participants were recruited from areas surrounding three sites: State College, PA (*N* = 167), Harrisburg, PA (*N* = 81), and Newark, NJ (*N* = 109).

### Race and Ethnicity

**Figure 3:**
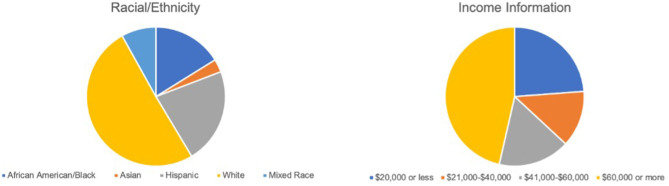
LAnTs sample demographics, including racial/ethnic background (**left**) and income (**right**).

Caregivers identified 58 of the infants (16%) as African American/Black, 9 (3%) as Asian, 78 (22%) as Latinx, 180 (50%) as white, and 27 (8%) as mixed race. Five (1%) additional caregivers declined to provide this information (see Figure 3, left).

### Annual Household Income

Across the sample, 49 families (14%) reported a household income of $15,000 or less, 20 (6%) reported $16,000–20,000, 22 (6%) reported $21,000–30,000, 16 (5%) reported $31,000–40,000, 22 (6%) reported $41,000–50,000, 29 (8%) reported $51,000–60,000, and 140 (39%) reported an income above $60,000. Fifty-nine (17%) additional caregivers declined to provide this information (see Figure 3, right).

### Parental Education

For mother's education, 11 (3%) completed grade school only, 17 (5%) had some high school, 36 (10%) graduated from high school, 57 (16%) had some college or trade/technical degree, 73 (20%) were college graduates, 58 (16%) had graduate training, and 66 (19%) had a graduate degree; 39 (11%) additional caregivers declined to provide this information. For fathers, 11 (3%) completed grade school only, 15 (4%) had some high school, 50 (14%) graduated from high school, 60 (17%) had some college or trade/technical degree, 70 (20%) were college graduates, 42 (12%) had graduate training, and 56 (16%) had a graduate degree; 53 (15%) additional caregivers declined to provide this information.

### Infant Temperament

**Figure 4:**
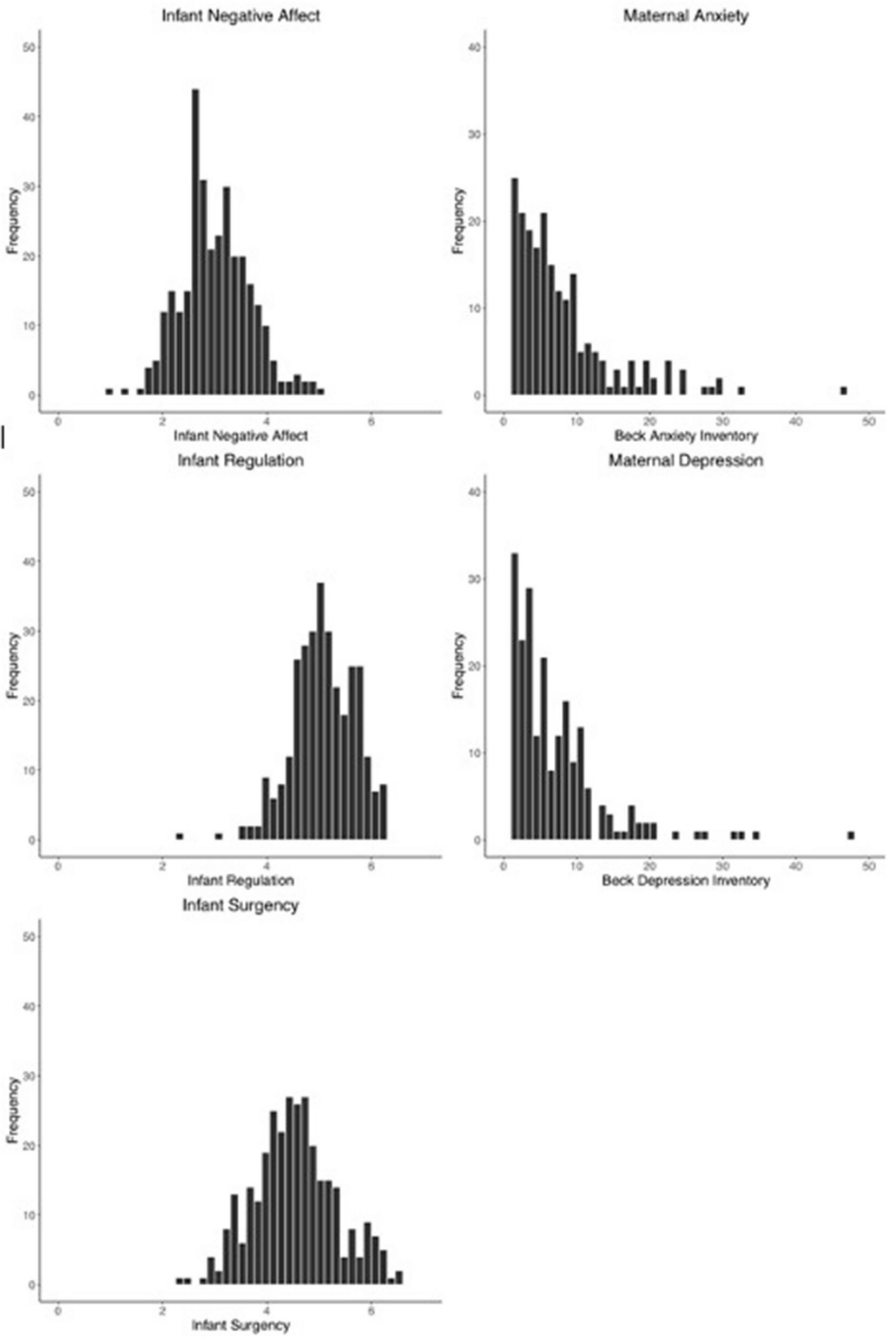
Histograms noting the distribution of core measures of infant temperament for the higher-order scales of negative affect, regulation, and surgency from the Infant Behavior Questionnaire (**left column**) and maternal symptoms of anxiety and depression (**right column**) from the Beck Anxiety and Beck Depression Inventories, respectively, at the point of enrollment.

Of our enrolled families, 312 parents completed the Infant Behavior Questionnaire (39) (one parent did not provide data for the negative affect subscale). For high-order factors, infants were rated on negative affect (*M* = 3.01, *SD* = 0.66, Min = 1.00, Max = 5.08), surgency (*M* = 4.50, *SD* = 7.84, Min = 2.37, Max = 6.53), and orienting/regulation (*M* = 5.08, *SD* = 6.09, Min = 2.28, Max = 7.00; see Figure 4) at time of enrollment.

### Parent Psychopathology

**Figure 5:**
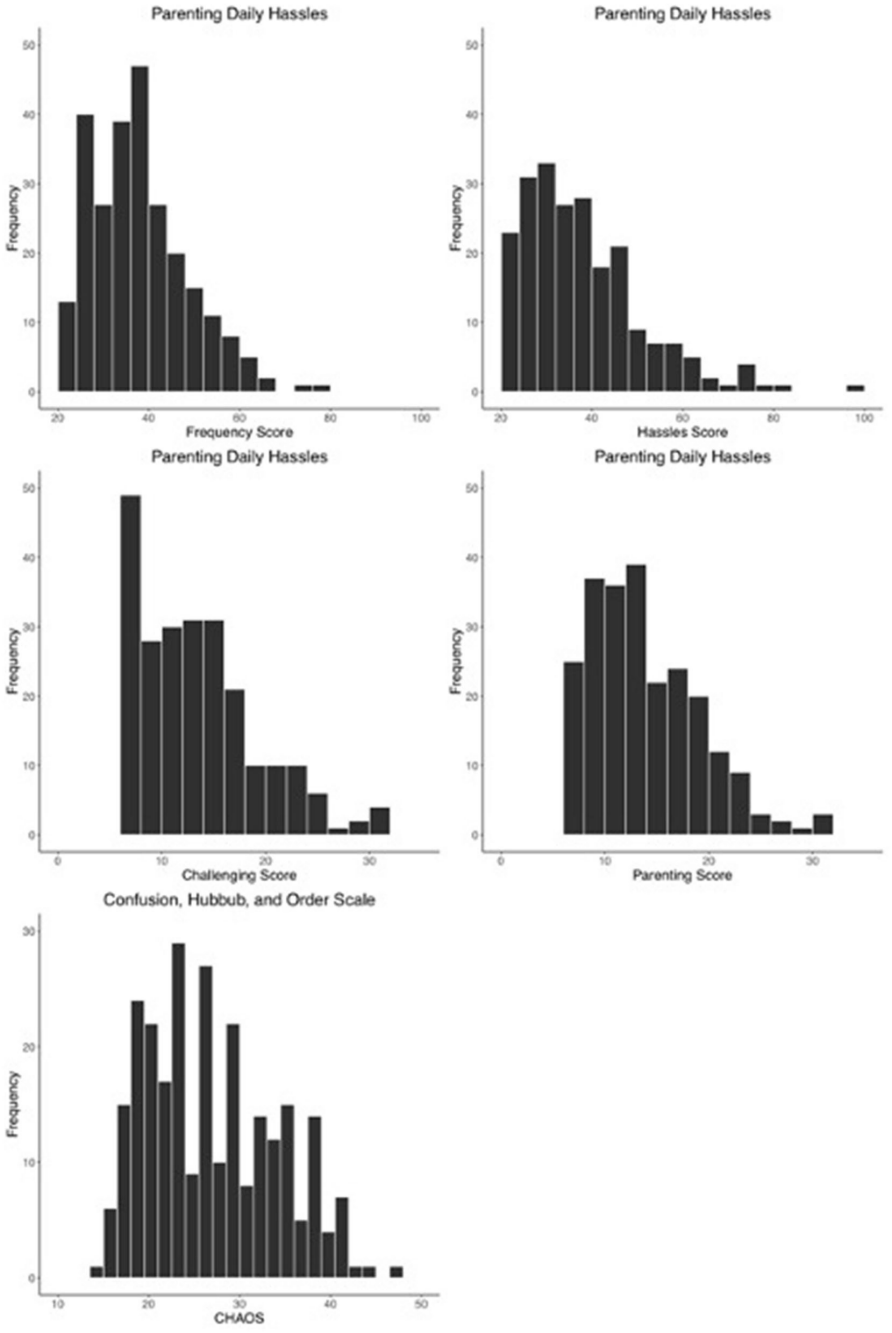
Histograms noting the distribution of core measures of parental perception of the environment at the point of enrollment. The first two rows present scores from of Parenting Daily Hassles with the frequency, hassles intensity, challenging behaviors, and parenting subscales. The third row presents the distribution of scores from the Confusion, Hubbub, and Order Scale.

Parents completed the Beck Anxiety Inventory (BAI) (40) and the Beck Depression Inventory (BDI) (41) as measures of parental psychopathology (see Figure 5). Values were prorated to account for missing values, such that the denominator of the sum score was adjusted for each item a parent did not complete on the questionnaire. Of our enrolled families, 272 parents completed the BAI at the time of enrollment (*M* = 6.66, *SD* = 7.55, Min = 0.00, Max = 53.00). The BDI was completed by 277 parents at the time of enrollment (*M* = 5.80, *SD* = 6.49, Min = 0.00, Max = 48.00).

### Home Life and Parenting

As an assessment of environmental confusion in the home, 265 parents completed the Confusion, Hubbub, and Order Scale (CHAOS) (42) (*M* = 27.18, *SD* = 7.25, Min = 15.00, Max = 50.00; Figure 5). Parents also completed the Parent Daily Hassles Survey (PDHS-R), an assessment of the frequency and intensity of daily hassles (43). At enrollment, 263 parents completed the frequency of hassles scale (*M* = 37.09, *SD* = 13.76, Min = 20.00, Max = 100.00) and 235 parents completed the intensity of hassles scale (*M* = 37.09, *SD* = 13.76, Min = 20.00, Max = 100.00). The PDHS-R further provides a challenging behavior and parenting task intensity score. The challenging behavior total score is obtained by summing seven items from the intensity scale scores and the parenting tasks scale is obtained by summing eight items from the intensity scale. At enrollment, 234 completed the challenging behavior subscale (*M* = 13.99, *SD* = 5.89, Min =7, Max = 35.00) and 233 parents completed the parenting task intensity subscale (*M* = 14.39, *SD* = 5.17, Min = 8.00, Max = 32.00).

## Discussion

The LAnT study's multi-method approach aims to (1) test the three models proposed by Field and Lester (17) and (2) examine the association between early patterns of attention to threat and BI at age 2 (3). This work fills evident gaps in the literature since the attention-affect research (1) has focused on adult clinically-defined populations, (2) often does not systematically assess constructs across multiple tasks and contexts, and (3) rarely takes a *developmental* view that examines core mechanisms as they emerge in infancy in hopes of differentiating between normative patterns and patterns associated with specific risk trajectories. This line of research reflects calls from the National Institute of Mental Health (NIMH) to implement the Research Domain Criteria (RDoC) across processes and across time (44). Here, we integrate multilevel mechanisms by examining response to potential threat (negative valence systems), attention patterns (cognitive systems) and early patterns of affect across varying socioemotional contexts (negative valence systems and social processes). We also go to the heart of NIMH's Objective 2, by characterizing trajectories of neural and behavioral development in order to identify clinically useful indicators of change across illness trajectories. This approach also parallels emerging studies (45) that examine selective attention and responsiveness to emotional expression as a means of scaffolding the development of empathy and social cognition. The available data also suggest that attention patterns, and their associations with socioemotional functioning, may change over time (46–48). Thus, it will be important to continue longitudinal assessments beyond toddlerhood and into early childhood.

By capturing the earliest mechanisms that may be causally linked to a pervasive set of problem behaviors, the study applies innovative measurement techniques to central questions of socioemotional development and may shape future research. The systematic assessment of attention bias patterns, socioemotional profiles, and environmental characteristic will allow us to delineate the time course of any emerging interrelations. Finally, as outlined in the current paper, the measures generated through the protocol can serve as the foundation for numerous other questions of interest to the scientific community.

## Data Availability Statement

The datasets for this study will be shared in the National Institute of Mental Health Data Archive (NDA) and Databrary (49) as data are collected, processed, and curated. Inquiries regarding data sharing and the status of the data can be addressed to the study PIs.

## Ethics Statement

The studies involving human participants were reviewed and approved by the Institutional Review Boards at Pennsylvania State University and Rutgers University, Newark. Written informed consent to participate in this study was provided by the participants' legal guardian/next of kin.

## Author Contributions

KP-E, VL, KB, AF, and LAnTs team drafted the manuscript. KP-E, VL, KB, and AF conceptualized the study and wrote the grant funding the research. The LAnTs team designed the tasks, collected data, and wrote the protocol descriptions. All authors contributed to the article and approved the submitted version.

## The LAnTs Team

Lori Reider^2^, Jessica Burris^2^, Denise Oleas^2^, Anna Zhou^1^, Centia Thomas^1^, Samantha Leigh^1^, Brendan Ostlund^1^, Berenice Anaya^1^, Kelley Gunther^1^, Alicia Vallorani^1^, Elizabeth Youatt^1^, Caitlin Smith^1^, Norbert Promagan^1^, Kayla Brown^1^, Laura Bierstedt^2^, Claudia Pinzon^2^, Kali Revilla^2^, Michell Sarquez^2^, Piumi Rajasekera^1^, Elveena Fareedi^1^, Annika Kershner^1^, Meghan McDoniel^1^, Xiaoxue Fu^1^, Santiago Morales^1^, Leigha MacNeill^1^, Eran Auday^1^, Briana Ermanni^1^, Dara Tucker^1^, Kelly Metcalf^1^.

^1^ Department of Psychology, The Pennsylvania State University, University Park, PA, United States

^2^ Department of Psychology, Rutgers University, Newark, NJ, United States

## Conflict of Interest

The authors declare that the research was conducted in the absence of any commercial or financial relationships that could be construed as a potential conflict of interest.
