# Supplementary material for: Study Protocol: Longitudinal Attention and Temperament Study
Source: Front Psychiatry. 2021 Jun 8;12:656958. doi: 10.3389/fpsyt.2021.656958 (PMC8218812; doi:10.3389/fpsyt.2021.656958)
Supplement: Supplementary file 1 [file Data_Sheet_1.pdf]

Supplementary Material

Detailed LAnTs Measures

Demographic and Health Information

Pregnancy History. Caregivers reported on their child's weight at birth, indicated (yes or no) whether their child was born within 3 weeks of the due date, or whether there were any birth complications or serious health issues since birth. Finally, parents indicated (yes or no) whether mothers smoked, drank alcohol, or took any medication during pregnancy. If medication use was indicated, parents then noted the type and extent of usage. This measure was collected at the family's first visit.

Breastfeeding Questionnaire. Caregivers indicated (yes or no) whether they had ever breastfed the child, if they were currently breastfeeding the child, if the child had ever received formula, and if the child had started eating solid foods. If applicable, caregivers also indicated how old their child was (in months) when breastfeeding stopped, when they first received formula, and when they started eating solid foods. This measure was collected at the 4-, 8-, 12-, 18-, and 24-month time points.

Child Motor Milestones. Caregivers indicated when their child (age in months) was first able to: sit unsupported, crawl, pull to a standing position, stand momentarily without supports, show stepping movements with support, and walk independently. This measure was collected at the 4-, 8-, 12-, 18-, and 24-month time points.

Child Sleep Routine Questionnaire (CSRQ). For each day of the week, caregivers indicated when their child usually goes to sleep and wakes up (e.g., 7pm, 7am) and the average number of hours slept for weekday and weekend nights. A 'sleep need' index can be calculated by multiplying the average weekday sleep time by 5 and the average weekend sleep time by 2. This measure was collected at the 4-, 8-, 12-, 18-, and 24-month time points.

Child Care History Survey (CCHS). In this questionnaire, parents were asked to describe the various 'care arrangements' in place for their child; up to five care arrangements could be described. For each care arrangement, parents indicated (1) who provides the care (e.g., child's father, child's grandparent, Head Start), (2) whether other children (including siblings) are being cared for (yes/no), (3) when the arrangement began (month/year), (4) how many days per week on average the child spent in the arrangement, and (5) how many hours per day the child was usually in the arrangement. The total number of days per week and hours per day the child spent in non-parental childcare are calculated by summing across arrangements. This measure was collected at the 4-, 8-, 12-, 18-, and 24-month time points.

Family Size Questionnaire. To assess family structure, parents were asked: 1. Including yourself, how many people live in your home?, 2. Of the people living in your home, how many are under 18?, 3. Has the number of people living in your home changed in the last year?, 4. If so, how many total people lived in your home one year ago? This measure was collected at the 12-, 18,- and 24-month time points.

Eye-Tracking Tasks

Eye tracking data were collected across sites using SMI eye tracking systems, either the SMI RED or REDm system, both offering comparable specifications/capabilities (SensoMotoric Instruments, Teltow, Germany). Participants were seated ~60cm from a 22" Dell monitor for stimulus presentation, in a high chair. If needed, infants could also sit on their parent's lap or on

the lap of an experimenter. Gaze was calibrated using a 5-point calibration followed by a 4-point validation, using an animated flower on a black screen and infant-friendly music. Gaze data were sampled at 60 Hz and collected by Experiment Center (SensoMotoric Instruments, Teltow, Germany). Infants/toddlers were calibrated below of 4° of visual angle from all calibration points.

Adults completed two tasks, the Parent Dot-Probe Task and two blocks of the Matrix Task, one with face stimuli and one with animal stimuli. All adult tasks recorded eye tracking information as well as button press responses. Infants completed three eye tracking tasks: the Infant Dot-Probe, Vigilance, and Overlap tasks [1]. Order of presentation for both adults and infants was randomized prior to the visits, with one exception--both blocks of the adult Matrix task were always presented together, with a randomized presentation order.

Infant Dot-Probe. An adaptation of the classic adult Dot-Probe task, designed to assess attentional biases [2], was presented to infants, relying on gaze instead of button presses to assess attention biases. Consistent with the calibration procedure, stimuli were presented using Experiment Center. Infants were presented with up to 60 experimental trials, ending either when all trials were completed or when the infant could no longer attend to the task. Each trial was initiated when the infant's attention was on a video clip presented centrally on the screen, which was triggered either when the infant fixated for at least 100ms or when the experimenter determined that the infant was looking at the video clip. If the participant did not attend to the center of the screen, the slide advanced after 10000ms. Next, a horizontal pair of faces sampled from the NimStim face set appear on the screen for 1000ms [3]. Pairs of faces were either angry and neutral, happy and neutral, or two neutral faces, of the same actor. Eight actors (4 male) provided neutral, happy, or angry, closed mouth images. Facial stimuli were approximately 19 cm x 13cm and the visual angle of each face was 17.99° (H) x 12.37° (W). The faces were 24.45° apart. (~26cm from center)

Following each pair of faces, a 4cm asterisk "probe" (3.82° visual angle) appeared on the screen in the same location as either the left or right face for 500ms. If the asterisk appeared in the same location as the "emotional" (i.e. angry or happy) face, the trial was marked as "congruent." Conversely, if the asterisk appeared in the same location as the neutral face, the trial was marked as "incongruent." This generated a total of 5 different trial types: angry congruent, angry incongruent, happy congruent, happy incongruent, or neutral. Finally, the trial was concluded with a 1000ms ITI, which was a blank screen. No consecutive trials were identical.

Areas of interest (AOIs) were drawn as ellipses enclosing both the faces and the probes. A 2cm "error margin" was added to each ellipse, to account for the deviation permitted in the calibration procedure. Analyses were based on gaze to these designated AOIs. Fixations, defined as gaze maintained for at least 80ms within a 100-pixel maximum dispersion, were extracted with BeGaze (SensoMotoric Instruments, Teltow, Germany). All other computations of gaze metrics were performed using in-house R scripts.

Variables of interest included dwell to each facial stimulus (for the purposes of this task, dwell is defined as both fixations to an AOI as well as saccades within an AOI) as well as latency to fixate on the probe. The latency to fixate on the probe was designed to emulate the latency to press a button that is often utilized in adult iterations of the Dot-Probe task [4]. An average dwell to each face was computed for each pairing of emotional stimuli, and an average latency to probe value was computed for each trial type.

Metrics were cleaned on a trial-by-trial level. If gaze was not detected on the screen during a trial, the trial was not included in dwell time calculations. Additionally, if a fixation was not

detected to the probe during a trial, that trial was not included in latency calculations. Dwells/latencies from trials deemed valid by these criteria were then averaged across trial type.

With these metrics, a number of potential analyses can be carried out. For example, we can generate proportion scores to assess preferential looking to the facial stimuli. Proportions are computed for angry and happy faces by taking the average dwell time to each emotional face and dividing it by the sum of average dwell to that face and the average dwell to the paired neutral face. Bias scores are computed for angry and happy faces by subtracting the average fixation time to the neutral face from the average fixation time to the emotional face. Bias scores can also computed using the average latency-to-probe values. Here, bias scores for happy and angry faces are computed as average latency to incongruent trials minus the average latency to congruent trials.

The dot probe traditionally makes the assumption that gaze is oriented toward the emotional face in labeling congruent and incongruent trials. Alternatively, for additional analysis, we can use the eye tracking data to re-label trial types on a trial-by-trial basis according to which face the participant was looking at just prior to probe onset as in Pérez-Edgar et al. [4]. In other words, trials were defined by participant gaze rather than a priori. For example, a gaze-defined trial was categorized as angry incongruent if the infant dwelled to the angry face just prior to the onset of the probe in the opposite location [4]. With these new trial classifications, data were processed once again as per the specifications detailed above.

Infant Overlap. Infants completed a version of a classic overlap task [5,6] to assess infants' ability to disengage from emotional faces. Consistent with the calibration procedure, stimuli were presented using Experiment Center. Infants were presented with up to 30 experimental trials, ending either when all trials were completed or when the infant could no longer attend to the task. Each trial was initiated when the infant's attention was on a video clip presented centrally on the screen, which was triggered either when the infant fixated for at least 100ms or when the experimenter determined that the infant was looking at the video clip. If the participant did not attend to the center of the screen, the slide advanced after 10000ms. Following was a central face sampled again from the NimStim face set for 1000ms [3]. Ten actors (5 male) provided neutral, happy, or angry, closed mouth images. Facial stimuli were approximately 12 cm x 8cm and the visual angle of each face was 11.42° (H) x 7.63° (W). Following the presentation of the face, a checkerboard stimulus then appeared in either the left or right periphery of the screen adjacent to the face (20.78° visual angle) for 3000ms. The checkerboard was 12 cm x 2.5cm, 11.42° x 2.39° visual angle. This progression of stimuli was concluded with a 1000ms ITI, which was a blank screen. No consecutive trials were identical in terms of face and probe placement.

Areas of interest (AOIs) were drawn as ellipses enclosing the face and rectangles enclosing the checkerboards. A 2cm "error margin" was added to each ellipse, to account for the deviation permitted in the calibration procedure. Analyses were based on gaze to these designated AOIs. Fixations, defined as gaze maintained for at least 80ms within a 100-pixel maximum dispersion, were extracted with BeGaze (SensoMotoric Instruments, Teltow, Germany). All other computations of gaze metrics were performed using in-house R scripts.

We computed dwell to the central face while the checkerboard stimulus was present, mirroring the analysis conducted by Morales and colleagues [5]. Similarly, we computed dwell to the checkerboard stimulus while the face was present [1]. For base processing, dwell is defined as the duration of fixations as well as saccades within the designated AOI. Sums and averages of these values for all trials in which gaze was tracked were generated for each emotion. We also computed the latency for the infant to fixate on the checkerboard stimulus.

Metrics can be cleaned on a trial-by-trial level. This included noting if gaze is detected on the screen during a trial, if a fixation is detected to the checkerboard stimulus during a trial, or noting if the infant fixated on the central face before fixating on the checkerboard stimulus. Averages for these metrics can be generated for each emotion. Trials with anticipatory eye movements in which latency to fixate on the checkerboard was less than 200ms are removed from the analysis [7]. After this cleaning, means and medians for each metric are calculated for each emotion.

Infant Vigilance. Eye-tracking data were collected during an infant vigilance task to assess infants' ability to detect emotional faces [8]. The task had 90 trials, with each trial beginning with a randomly presented fixation-dependent attention getting Baby Sensory animated videos with a black background and classical music dubbed in. Each trial was initiated when the infant's attention was on a video clip presented centrally on the screen, which was triggered either when the infant fixated for at least 100ms or when the experimenter determined that the infant was looking at the video clip. If the participant did not attend to the center of the screen, the slide advanced after 10000ms. Each trial continued with a random presentation of a face stimulus in one of the four corners of the screen. Faces were sampled from the NimStim face set and appeared for up to 4000ms or until the participant fixated it for 100ms [3]. Ten actors (5 male) provided neutral, happy, or angry, closed mouth images. Facial stimuli were approximately 9.50 cm x 6.50 cm and the visual angle of each face was 9.05° (H) x 6.20° (W). Faces were approximately 16.59° visual angle from center. No face stimuli appeared in the same location consecutively, and the order of face stimuli were randomized across participants. Location of the faces was counterbalanced across the four corners of the screen. There were 4000ms white screens that were shown after every 7th trial to minimize habituation and predictive looking. Task design and recording were completed by Experiment Center (SensoMotoric Instruments, Teltow, Germany).

The raw (X,Y) position of fixations were exported from BeGaze (SensoMotoric Instruments, Teltow, Germany). An area of interest (AOI) encircling and including the entire face stimulus was created and exported from BeGaze. A 2cm "error margin" was added to each ellipse, to account for deviation permitted in the calibration procedure. Data processing was restricted to gaze data within the face AOI. An in-house processing script was written in R version 3.6.2 (R Development Core Team, 2019) to measure latency to fixate the face AOI on each trial.

Metrics were cleaned on a trial-by-trial level. If a fixation was not detected to the face during a trial, that trial was not included in latency calculations. Furthermore, trials with anticipatory eye movements in which latency to fixate on the face was less than 200ms were removed from the analysis [7]. After this cleaning, average and median latencies were calculated for each emotion.

Adult Dot-Probe. An adaptation of the standard Dot-Probe task, designed to assess attentional biases [2], was presented to the infants' caregivers, relying on both gaze and button presses to assess attention biases. Eye tracking data were collected across sites using SMI eye tracking systems, using either the SMI RED or REDm system, both offering comparable specifications/capabilities (SensoMotoric Instruments, Teltow, Germany). Participants were seated ~60cm from a 22" Dell monitor for stimulus presentation. Gaze was calibrated using a 5-point calibration followed by a 4-point validation. Gaze data was sampled at 60 Hz and collected by Experiment Center (SensoMotoric Instruments, Teltow, Germany). Adults were calibrated below 2° of visual angle deviation from all calibration points.

Consistent with the calibration procedure, stimuli were presented using Experiment Center. Adults were presented 160 experimental trials. Each trial began with a centrally-presented fixation

cross for 500ms. Following was a horizontal pair of faces sampled from the NimStim face set [3] for 500ms. Pairs of faces were either angry and neutral, happy and neutral, or two neutral faces. Facial stimuli were approximately 19 cm x 13cm and the visual angle of each face was 17.99° (H) x 12.37° (W). The faces were 24.45° apart. Eight actors (4 male) provided neutral, happy, or angry, closed mouth images.

Next, a "probe" appeared on the screen in the same location as either the left or right face for 500ms. The probes were two dots that were either horizontally or vertically. Participants were prompted to press the "D" button on a keyboard if the dots were horizontal, and the "K" button if the dots were vertical. If the probe appeared in the same location as the "emotional" (i.e. angry or happy) face, the trial was marked as "congruent." Conversely, if the probe appeared in the same location as the neutral face, the trial was marked as "incongruent." This generated a total of 5 different trial types: angry congruent, angry incongruent, happy congruent, happy incongruent, or neutral. Finally, the trial was concluded with a 1000ms ITI, which was a blank screen.

Areas of interest (AOIs) were drawn as ellipses enclosing both the faces and the probes. A 1cm "error margin" was added to each ellipse, to account for the 1° of deviation permitted in the calibration procedure. Analyses were based on gaze to these designated AOIs. Fixations, defined as gaze maintained for at least 80ms within a 100-pixel maximum dispersion, were extracted with BeGaze (SensoMotoric Instruments, Teltow, Germany). All other computations of gaze metrics were performed using in-house R scripts.

Variables of interest included total fixation duration to each facial stimulus, latency to fixate on the probe, and latency to button press. An average dwell to face was computed for each pairing of emotional stimuli. An average latency to probe, as well as reaction time (RT) to button press, was computed for each trial type. For button press data, only correct trials were used in computing average and medians for latencies.

Metrics were cleaned on a trial-by-trial level. If fixations were not detected on the screen during a trial, the trial was not included in dwell time calculations. Additionally, if a fixation was not detected to the probe during a trial, that trial was not included in latency calculations. Finally, RT latencies less than 150ms and greater than 2000ms were excluded from calculations [9-11]. Dwells/latencies from trials deemed valid by these criteria were then averaged across trial type.

With these metrics, one can generate proportion scores to assess preferential looking to the facial stimuli. For example, proportions can be computed for angry and happy faces by taking the average fixation time to each emotional face and dividing it by the sum of average fixation time to that face and the average dwell to the paired neutral face. Bias scores can be computed for angry and happy faces by subtracting the average fixation time to the neutral face from the average fixation time to the emotional face. Bias scores for happy and angry faces can also be computed as average latency to incongruent trials minus the average latency to congruent trials. Bias scores are generated for both fixation data and button-press data.

The dot probe traditionally makes the assumption that gaze is oriented toward the emotional face in labeling congruent and incongruent trials. Alternatively, for additional analysis, we used the eye tracking data to re-label trial types on a trial-by-trial basis according to which face the participant was looking at just prior to probe onset as in Perez-Edgar et al. [4]. In other words, trials were defined by participant gaze rather than a priori. For example, a gaze-defined trial was categorized as angry incongruent if the adult fixated to the angry face just prior to the onset of the probe in the opposite location [4]. With these new trial classifications, data were processed once again as per the specifications detailed above.

Adult Vigilance. An adaptation of the classic adult visual search task, designed to assess rapid attention to threat [12], was presented to the infants' caregivers, relying on both gaze and button presses to assess attention biases. Adults completed two blocks of the task in a random order. The face block consisted of 72 trials in which a 3x3 matrix of emotional faces presented to the participant. There were 4 trial conditions: angry target-present (24 trials), angry target-absent (12 trials), happy target-present (24 trials), and happy target-absent (12 trials). In the angry target-present trials there were eight happy faces and one angry target face; in the angry target-absent trials there were 9 happy faces, and vice versa for the happy target trials. Twenty-four faces from the NimStim stimulus set [3] were used, each appearing once as the target in an angry target-present trial and once as the target in a happy target-present trial. Within the 3x3 matrix, each face had a width of 15% of the screen and a height of 33% of the screen, with images directly adjacent to one another and white bars present on the left and right side of the matrix. Trial order, target position, and distractor identities and positions were all randomized. Participants were instructed to press the "J" key if all stimuli were from the same category or the "F" key if one of the images were from a different category. Participants then pressed the space key to advance each trial after responding.

The animals block consisted of 72 trials, each with a 3x3 matrix of pictures of snakes and frogs presented to the participant. There were 4 trial conditions: snake target-present (24 trials), snake target-absent (12 trials), frog target-present (24 trials) and frog target-absent (12 trials). In the snake target-present trials there were eight frogs and one snake target; in the snake target-absent trials there were 9 frogs, and vice versa for the frog target trials. Twenty-four images of animals were used, each appearing once as the target in a snake present trial and once as the target in a frog present trial. Within the 3x3 matrix, each animal image had a width of 25% of the screen and a height of 33% of the screen, with images directly adjacent to one another and white bars present on the left and right side of the matrix. Trial order, target position, and distractor images and positions in the matrix were all randomized. Participants were instructed to press the "J" key if all stimuli were from the same category or the "F" key if one of the images were from a different category. Participants then pressed the space key to advance each trial after responding.

Areas of interest (AOIs) were drawn as squares enclosing each separate animal image in the matrix. All analyses were based on gaze to these designated AOIs. Latency to visually fixate the target on target-present trials, as well as accuracy and reaction time of button press responses were extracted with BeGaze software (SensoMotoric Instruments, Teltow, Germany).

Behavioral Tasks

4-Month Reactivity. At the 4-month visit, infant temperamental reactivity was assessed behaviorally with a validated reactivity battery [13,14]. The infant was seated in a car seat with a primary caregiver seated nearby out of the infant's line of sight. The experimenter then played the infant two audio tracks, one consisting of a series of sentences with overlapping voices and a second containing three groups of ten repeated syllables. Interwoven with the audio tracks, the experimenter presented the infant with a series of mobiles. The mobiles consisted of plush neutral figures (bears or jungle animals, with order counterbalanced). The figures were presented in phases, going from one to three to five figures. Each phase lasted 20 seconds.

Lab-TAB. The Laboratory Temperament Assessment Battery (Lab-TAB; [15]) is a standardized assessment of infant responsiveness to stimuli in a controlled setting, aimed at eliciting emotional and behavioral reactions to stimuli. Infants completed the Lab-TAB at the 8-, 12-, 18-, and 24-month time points. The specific reactions of interest vary along 5 broad

dimensions of infant temperament: activity level, anger proneness, fearfulness, interest/persistence, and joy/pleasure. While the full Lab-TAB manual provides 20 episodes, we selected 6 episodes for each timepoint that best address the research question of interest. For the non-locomotor 8- and 12-month assessments, infants completed the stranger approach, puppet, peekaboo, container, robot, and arm restraint tasks. All tasks were completed with the infant seated in the high-chair unless the infant was unwilling to sit in the highchair and was then placed on the caregiver's lap. For the locomotor 18- and 24-month assessments, the same tasks were completed with one exception—the robot task was replaced with the spider task. All tasks for locomotor infants were completed with the infant seated unrestrained in a small chair unless the infant was unwilling to sit in the chair, and was then placed on the caregiver's lap. While some adjustments were made to accommodate space restrictions, the Lab-TAB guidelines were followed at each testing site. Instructions for the caregiver were provided in the caregiver's preferred language (e.g., English or Spanish). All other language used for each episode (i.e. scripts for the infant) was provided in English.

Stranger Approach. During the stranger approach episode, the infant was seated at one end of the room facing the entryway where the stranger entered the room. Unlike the original protocol [15], the sex of the stranger was not controlled due to staffing and scheduling limitations. However, all research assistants wore identical black sweaters and a black cap with upswept hair, which helped obscure individual features. The caregiver was in the room standing behind the infant's view, and was instructed not to interact with the child. At the start of the task, the stranger appeared at one end of the room and waited 15 seconds, maintaining neutral expression and eye contact with the infant for the duration of the task. The stranger then said, "Hello [infant name], I'm going to come a little closer to you" and proceeded to approach the infant for 10 seconds, stopping halfway from the infant. The stranger then paused for 15 seconds and said, "I'm going to come a little closer to you", and approached the infant for another 10 seconds until the stranger was close to the infant. The stranger then kneeled down in front of the infant for 15 seconds. In the locomotor version of Stranger Approach, the stranger changed their trajectory to follow the child if the child moved around the room.

Puppet. In the puppet episode, the infant was seated at a table with the researcher and caregiver on each side of the infant. The researcher put on two exciting hand puppets and read through the puppet show script. The researcher used the hand puppets to talk to and tickle one another while maintaining a neutral face and not making eye contact with the infant. At the end of the puppet show, the infant was asked "would you like to touch the puppets?", and was given 10 seconds to do so. The task was complete when the infant touched the puppets or after 10 seconds if the infant did not touch the puppets.

Attractive Toy (Container). During the container episode, the infant was seated at a table with the researcher and caregiver on each side of the infant. The researcher gave the infant an exciting toy (a colorful, vibrating, sound making toy pig) to play with for about 30 seconds. The researcher then removed the toy from the infant and placed the toy in a clear plexiglass container, latched the lid shut, and pushed the container with the toy towards the child. The container remained in front of the child for 30 seconds. The researcher then removed the toy from the container and allowed the child to play with the toy. This was repeated up to 3 times (i.e. 3 trials were attempted if possible). At the end of the final trial, the infant was asked "would you like to touch the pig?", and was given 10 seconds to touch the toy pig. The task was complete when the infant touched the toy. The task was terminated if the infant did not touch the toy within 10 seconds.

Peekaboo. During the peekaboo episode, the infant was seated at a table with the caregiver on one side of the infant, and a small stage on the other side of the infant. The researcher went behind the stage and initiated a game of peekaboo for 30 seconds. The researcher peeked his/her head out from the curtain in random locations and said "peekaboo!", smiling and remaining visible to the infant for about 2 seconds before returning behind the curtain. The researcher waited 5 seconds behind the curtain and then appeared from the curtain in a different location. This continued for approximately 30 seconds, and the infant viewed 6 instances of peekaboo. After the last instance of peekaboo, the researcher kneeled behind the stage out of the infant's site for 10 seconds before ending the task.

Unpredictable Mechanical Toy (Robot) for Non-Locomotor Infants. During the robot episode, the infant was seated at a table with the researcher and caregiver on each side of the infant. The researcher placed a loud, flashing, unpredictable mobile toy robot at one end of the table. The researcher used a remote control to move the toy toward the child until the toy was just within the child's reach. The robot paused at this closest point for 10 seconds, and was then reset to the starting point for 5 seconds. At the end of the third trial, the robot turned right and then left once the robot reached the closest point to the child. Three trials were attempted if possible. At the end of the final trial, the infant was asked "would you like to touch the robot?", and was given 10 seconds to touch the robot. The task was complete when the infant touched the robot. The task was terminated if the infant did not touch the robot after 10 seconds.

Unpredictable Mechanical Toy (Remote Controlled Spider) for Locomotor Infants. During the spider episode, the infant was initially seated on the caregiver's lap in a chair at one end of the room facing the entryway. A large mechanical toy spider appeared in the entryway, and paused for 10 seconds. Then, the researcher used a remote control to move the toy spider toward the child until the spider was about halfway to the infant, and paused for 10 seconds. The researcher then moved the spider backward to its starting position and waited 10 seconds. The spider approach procedure was then repeated. If the infant moved around the room, the spider would change its trajectory to follow the child. Finally, the researcher brought the toy spider to the infant and asked, "would you like to touch the spider?" The infant was given 10 seconds to touch the spider. The task was complete when the infant touched the spider or 10 seconds, if the infant did not touch the spider.

Arm Restraint. In the arm restraint episode, infants were shown an interesting perpetual motion toy and given the opportunity to play with the toy. The researcher then prompted the caregiver to hold the infant's arms to restrain the infant from reaching or playing with the toy. The procedure lasted 30 seconds and two trials were attempted if possible. Between the first and second trial (about 30 seconds), the infant was allowed to play with the toy or generally return to baseline. Infant struggle during each trial was also coded only during the two, 30 second trials.

<u>Mother-Child Free Play.</u> Mothers and children were observed in the laboratory during a 5-minute free-play task at the 8-, 12-, 18-, and 24-month assessments. Mothers were instructed to play with their infants as they usually would at home, using a basket of toys which included balls, little animals, and trucks. The free-play session encompassed a *semi-structur*e and an *unstructured* component. The experimenter instructed mothers to begin the task by introducing a mildly-stimulating toy/object to their infant (semi-structured component), and to then freely play with the other toys as desired for the remainder of the task (unstructured component). The order of the semi-structured and unstructured components was standard across all families.

For the semi-structured portion of the task, the toy/object was selected based on level of stimulation (e.g., surprise, lighting, vibration), to capture temperament-linked individual differences in infants' fear responses, and observable maternal strategies to mitigate infants' negative affect. We employed two different stimulating toys across the study in order to maximize the novelty effect and the developmental sensitivity of the toys. At the 8- and 12-month visits, mothers introduce a Jack-in-the-Box to their infants as part of the semi-structured component. The Jack-in-the-Box had a spinning handle, which would play a tune until the pop-up toy came up as a surprise. Mothers were instructed to spin the Jack-in-the-box handle to play the entire tune until the surprise pop-up occurred at least once. The end of the semi-structured component was marked once mothers completed at least one cycle of the Jack-in-the-Box pop-up. At the 18- and 24-month visits, mothers introduced a HoMedics Ribbit mini massager as the stimulating toy for the semi-structured component. The mini massager was a lightweight, hand-held device, roughly the size of an adult's hand. The device had four leg-extensions, which vibrated and were illuminated with a button-press. Mothers were instructed to introduce the mini massager by 1) showing the toy to their child, 2) pulsing the on-button three times for the toy to vibrate and light-up briefly, and 3) fully pressing the on-button for the device to stay on. In this case, the end of the semi-structured component was marked once mothers completed all three steps or the child took possession of the toy.

Social Dyad Visit. The social dyad was completed at the 24-month assessment as a measure of behavioral inhibition in the infant [13,16]. Dyads were paired based on sex. When possible, we also ensured that we did not pair infants that were both at the extreme of temperamental fearfulness or exuberance. To determine the distribution of temperamental fear and exuberance we first examined the distribution of ITSEA scores for infants 11 to 19 months across two unique samples [8,17]. As in previous work [18], we focused on the subscales of inhibition to novelty (*M* = 0.88, *SD* = 0.48), separation distress (*M* = 0.88, *SD* = 0.40) and anxiety/worry (*M* = 0.22, *SD* = 0.25) to identify normative ranges of fearful temperament. To identify normative ranges of exuberant temperament, we focused on the activity/impulsivity (*M* = 0.72, *SD* = 0.39), inhibition to novelty, and depression withdrawal subscales (*M* = 0.05, *SD* = 0.10). In the current sample, we used +/- 1SD cutoffs identified from the archival data to identify extreme temperamental profiles. Infants who met at least two of the three following criteria were deemed temperamentally fearful: ITSEA inhibition to novelty score ≥ 1.36, ITSEA separation distress score ≥ 1.28, ITSEA anxiety/worry score ≥ 0.47. Infants who met at least two of the three following criteria were deemed temperamentally exuberant: ITSEA activity/impulsivity score ≥ 1.11, ITSEA inhibition to novelty score ≤ 0.40, ITSEA depression/withdrawal score of 0. When possible, we used ITSEA values reported during the 18-month timepoint. When ITSEA data were not available for the 18-month timepoint, we relied on 12-month or 24-month reports.

At the beginning of the assessments, families were brought to separate rooms and completed informed consent procedures. RSA electrodes were attached to the infants and a 4-minute baseline was completed (see *RSA*). Researchers then explained to parents that they would be going into another room where their infant would meet another infant and engage in a few tasks. Parents were asked that during this time they remain as neutral as possible. Additionally, they were provided the MacArthur-Bates Communicative Development Inventory Short Form (MB-CDI-SF) questionnaire to complete during this time to discourage engagement with their infants (see *Questionnaires*). Parents and infants were then brought to a new location for the dyad tasks.

Social Dyad Free Play Task. The free play task began as soon as the infants entered the shared space. The space was set up with a variety of toys available in the center with parents seated to the side of the room. The researcher also stood to the side of the space. Infants were allowed to play freely for 5 minutes.

Toy Share Task. The researcher had both infants sit in the center of the space and introduced them to a Mr. Potato Head doll, showing the infants how the various pieces could be put together. The researcher then gave one infant the Mr. Potato Head doll and the other infant the rest of the pieces and then moved to the side of the space. Infants were allowed to play as they wished for 2 minutes.

Cool Toy Task. The researcher had both infants sit in the center of the space and brought out a scooter covered in a blanket. The design of the scooter allowed for only one infant to play at a time. The scooter was particularly attractive as it also played music and sounds. The research told the infants they were going to show them a cool toy and then removed the blanket and moved to the side of the space. Infants were allowed to play as they wished for 2 minutes.

Behavioral Coding

4-Month Reactivity. Behavioral coding of reactivity focused on the infants' affective and motoric responses to the auditory and visual stimuli [13,19]. Coding captured the frequency of arm waves, arm wave bursts, leg kicks, leg kick bursts, back arches, hyper extensions, and smiles. Amount of time engaged in vocalizations, fusses, and crying was also coded. The presence (vs. absence) of hand clasping, finger/foot sucking, and feet rubbing were also noted.

Lab-TAB Coding. Videos of the episode were coded for the baseline state of the infant and the caregiver prior to the start of the task (10 seconds before task onset). A single overall rating prior to the presentation of the stimulus was used to characterize the baseline of the infant prior to the start of the task from: 1-Tired/drowsy, 2-Alert/calm, 3-Alert/active, 4-Fussy, or 5-Crying. A single overall rating was also made regarding the level of caregiver interference prior to the start of the task from: 0-Not interfering,1-Mild interference, or 2-interfering. Videos were also given an overall rating to indicate the effectiveness of the caregiver's behavior throughout the task (where applicable). Videos were coded continuously for facial affect, bodily fear and intensity of bodily fear, bodily sadness, escape behaviors, positive motor activity, self-soothing behavior, startle responses, hesitation to approach (18 and 24 months only), vocal valence and intensity of vocalization, general task engagement, reaching for stimuli, touching stimuli, and child location.

Mother-Child Free Play Coding. Observations were coded for maternal behavior, maternal control, maternal affect, infant behavior, and infant affect. All observations were coded continuously and were mutually exclusive to measure the frequency (in seconds) that mothers and children spent in different behavioral and emotional states across the entire task.

Social Dyad Free Play Coding. The 5-minute free play task was broken into 15 20-second epochs. For every infant, researchers coded each epoch for 9 behaviors all on 5-point scales. Codes included activity level (1 – low levels of movement to 5 – high levels of movement), social interest (1 – no interaction with dyad partner to 5 – full engagement with dyad partner), wariness (1 – no hesitation to 5 – high levels), unfocused behavior (1 – concentration on a singular activity to 5 – bouncing between multiple activities), negative affect (1 – no facial or vocal signals of negative affect to 5 – constant facial or vocal signals of negative affect), positive affect (1 – no facial or vocal signals of positive affect to 5 – constant facial or vocal signals of positive affect), interaction with researcher (1 – no direct interaction with the research to 5 – constant interaction with the researcher), interaction with parent (1 – no direct interaction with the parent to 5 – constant

interaction with the parent), and parent-initiated interactions (1 – parent does not engage infant to 5 – parent constantly engages infant). Each infant was coded separately on a 5-point scale.

Respiratory Sinus Arrhythmia (RSA)

Electrocardiograph (ECG) signal from the infant was continuously recorded during a resting state, and during the Lab-TAB episodes [15] at 8 to 24 months, and the social dyad at 24 months. Gelled sensors (stickers) were placed by the experimenter on the child's right collarbone, and lower left and right rib prior to baseline. Resting RSA was 4 minutes in duration and the infant was positioned on a parent's lap and given non-stimulating toys to keep them occupied. Parents were instructed to avoid social contact and keep as neutral as possible. A PDA was attached to the back of the highchair or the parent's chair. At the 18- and 24- month visits, the infant wore the PDA inside a small backpack to allow for locomotor activity.

ECG was sampled at a rate of 500ms using Mindware MW1000A PDA devices and BioLab system (Mindware Technologies, Ltd., Westerville, OH). Data were analyzed offline using the Mindware editing program Mindware HRV, Versions 3.1.4 and 3.1.5, which identified interbeat-intervals and detected physiologically improbable intervals using a validated algorithm [20]. Trained personnel visually inspected ECG data for R-peak and artifact identification. RSA was calculated in 30-second epochs using the 0.240 – 1.040 Hz power band. The mean RSA value from across all epochs per task was used for analyses. RSA reactivity was computed in two ways: by subtracting resting RSA from task RSA, and by regressing task RSA on resting RSA (i.e., a residualized change) which controls for resting levels. For difference scores, positive scores indicate RSA augmentation (increases in RSA during task) while negative scores indicate RSA suppression (decreases in RSA during task). For residualized change scores, positive scores indicate more RSA augmentation than expected based on the sample, and negative scores indicate more RSA suppression than expected based on the sample"

Resting-State Electroencephalograph (EEG)

EEG was continuously recorded at 32 Ag/AgCl active scalp electrodes during a four-minute baseline task at the 8-, 12-, 18-, and 24-month assessments . Each infant was fitted with the active electrode cap (Brain Products actiCAP) after measuring their head from nasion to inion. A small amount of conductive gel was applied to each electrode site prior to placing the cap on the infant's head. The experimenter dispensed additional gel as needed while gently abrading the scalp until impedance levels were below 10 kΩ or the infant became fussy. The baseline task was divided into four one-minute blocks during which the lights in the room were either on ("lights-on") or off ("lights-off") to imitate eyes open and eyes closed conditions, respectively. This approach has been used in prior work with pediatric samples [21]. Blocks alternated between lights-on and lights-off conditions (on, off, on, off). A video showing neutral images moving continuously across the screen was presented on a 17" computer screen to help maintain infant attention. We prioritized a subset of electrodes related to questions of interest to reduce participant burden and expedite the capping process. The following electrodes were selected: Fp1, Fp2, F3, F4, F7, F8, Cz, C3, C4, T7, T8, P3, P4, P7, P8, O1, and O2. EEG was amplified using the Brain Products' ActiCHamp system and digitized at 500 Hz.

EEG data were preprocessed offline using Brain Vision Analyzer 2 (Brain Products GmbH, Germany). Data were filtered using a zero-phase Butterworth infinite impulse response (IIR) filter with low and high cutoffs of 0.1 Hz and 40 Hz (12 db/octave), respectively, and a 60 Hz notch filter. EEG data were referenced online to Fz, and then re-referenced offline to the average of

P7/P8. Prioritizing particular electrodes limited our ability to meet certain assumptions of referencing schemes more commonly used in infant EEG research, such as the common average (e.g., insufficient number of electrode sites). P7/P8 were selected from the prioritized electrodes given that these sites were relatively far from scalp sites of interest, were not biased towards one hemisphere, and were minimally influenced by undesired sources of interference to the signal [22]. EEG data were segmented into 1s epochs and, with the exception of delta-beta coupling, these segments were created with a 50% overlap. These segments were then baseline corrected and inspected for artifacts (e.g., eye blinks). Artifacts were defined as voltage steps exceeding ± 50 µV/ms, maximum voltage difference of less than .50 µV within a 100ms interval or more than 150 µV within a 200ms interval, or amplitudes exceeding ± 200 µV. Trained research assistants visually inspected and removed the identified artifacts specific to the electrode(s) of interest, namely, F3, F4, C3, C4, Pz, P3 and P4 for delta/beta coupling; Fp1, Fp2, F3, F4, F7, and F8 for frontal asymmetry; Cz for the aperiodic exponent. Spectral power at each frequency was calculated via a Fast Fourier Transformation (FFT) that utilized a Hamming window with 50% overlap. Power values were then exported for additional processing in R v3.6.1 (R Core Team, 2019). The following frequency windows were used in the calculation, except in the case of the aperiodic exponent: Delta (0.5 - 2 Hz), Alpha (6 - 9Hz), and Beta (11 - 18 Hz).

Delta-Beta Coupling. We exported second-by-second EEG power for the delta (0.5-2 Hz) and beta (11-18Hz) frequency bands [23] as opposed to averaging across the time series, which has been the common approach in the existing literature [24]. Participants with less than 10 segments were excluded from the calculation of delta-beta coupling. Power values across target electrodes were log transformed and then averaged to create composites for the Frontal (F3, Fz, F4), Central (C3, Cz, C4), and Parietal (P3, Pz, P4) regions based on the 10-20 System of Electrode Placement [25].

Frontal Asymmetry. A child had to provide at least 60 seconds of artifact-free EEG data during either the lights-on or lights-off condition for a frontal asymmetry score to be calculated (Hill et al., 2020). Spectral power in the 6-9 Hz frequency range was natural log transformed for electrode channels Fp1, Fp2, F3, F4, F7, and F8. Frontal asymmetry scores were computed by first subtracting activation in the left frontal electrode from activation in the right frontal electrode (i.e., ln(right) – ln(left) = frontal asymmetry score) for specific electrode pairs, that is, Fp1/Fp2, F3/F4, and F7/F8. Frontal asymmetry was calculated based on the average of these scores for each condition. A negative frontal asymmetry score reflects greater activation in the right hemisphere relative to the left hemisphere [13,26].

Neural noise. Neural power spectra were parameterized from 1-20 Hz via the "fitting oscillations & one over f" (FOOOF) algorithm, available as a Python toolbox (https://github.com/foooftools/fooof). A detailed overview of this method is provided by Donoghue and colleagues [27]. In brief, this algorithm estimates and removes the aperiodic slope of the power spectral density based on an exponential fit in semi-log space. The algorithm then identifies and subtracts out periodic oscillatory activity via an iterative process based on Gaussian model fitting. Model fitting is repeated until the periodic components have been removed, at which point the slope is refit and overall model fit is determined [27]. The aperiodic exponent, our putative marker of neural noise, is χ in the $1/f^{\chi}$ formulation, which FOOOF uses in the exponential fit of the aperiodic slope.

Questionnaires

In most cases, the following questionnaires were administered via Qualtrics before each laboratory visit, although some were administered during the laboratory visit (e.g., for participants who did not have access to a computer, tablet, or smartphone), and others were completed after the laboratory visit. The only exception was the MacArthur Bates Communicative Development Inventory (MB-CDI) [28], which was administered during the social dyad. Versions of each questionnaire were available in both English and Spanish, and were administered based on the caregiver's first language.

Infant Behavior Questionnaire—Revised (IBQ-R). The IBQ-R is a 191-item survey designed to assess general patterns of behavior associated with temperament in infancy (3-12 months) [29,30]. Parents rated how often they observed a behavior in the past week at the 4-, 8-, and 12-month time points. Each item describes an infant behavior (e.g., *During feeding, how often did the baby lie or sit quietly?*) using a 7-point scale (never, very rarely, less than half the time, half the time, more than half the time, almost always, always). Parents are also given a "not applicable" response option for use when the infant has not been observed in the situation described. Each item loads onto one of 14 subscales: Activity Level, Distress to Limitations, Fear, Duration of Orienting, Smile/Laughter, High-intensity Pleasure, Low-intensity Pleasure, Soothability, Falling Reactivity, Cuddliness, Perceptual Sensitivity, Sadness, Approach, and Vocal Reactivity. Items from each subscale are averaged to obtain scale scores. Each scale, in turn, loads onto one of three broader factors (Surgency, Negativity, Orienting/Regulation). The IBQ-R has demonstrated good internal consistency, reliability, and validity, including correlations with laboratory observations [30-32].

Toddler Behavior Assessment Questionnaire (TBAQ). The TBAQ is a 120-item survey designed to assess general patterns of behavior associated with temperament in young children (2-3 years) [33]. It was collected at the 12-, 18-, and 24-month time points. Parents rated how often their toddler displayed a specific behavior in the past month using a 7-point Likert scale (1=never, 2=very rarely, 3=less than half the time, 4=half the time, 5=more than half the time, 6=almost always, 7=always). Each item loads onto one of 11 subscales (Activity Level, Anger, Appropriate Attention Allocation, Inhibitory Control, Interest, Object Fear, Perceptual Sensitivity, Pleasure, Sadness, Social Fear, Soothability). Items from each subscale are averaged to obtain scale scores. Goldsmith [33] reported high levels of convergence with various subscales of the IBQ.

Infant-Toddler Socioemotional Assessment (ITSEA). The ITSEA is a 200-item survey designed to assess multiple dimensions of social-emotional problems and competencies in 1- to 3-year-old children [34]. It was collected at the 12-, 18-, and 24-month time points. Parents described their child on a set of behaviors or attributes for their child (e.g., Sleeps through the night; Is stubborn) in the past month on a three-point scale (0=Not true/rarely, 1=somewhat true/sometimes, 2=very true/often). A 'No opportunity' code allowed parents to indicate that they have not had the opportunity to observe certain behaviors (e.g., peer interactions). Each item taps into one of three problem domains (Internalizing, Externalizing, Dysregulation) or a Competence domain. The Externalizing Problems factor is comprised of three subscales (Depression/Withdrawal, General Anxiety, Separation Distress, Inhibition to Novelty). The Internalizing Problems factor is comprised of four subscales (Depression/Withdrawal, General Anxiety, Separation Distress, Inhibition to Novelty). The Dysregulation factor consists of four subscales (Sleep, Negative Emotionality, Eating, Sensory Sensitivity). The Competence factor is comprised of 6 subscales (Compliance, Attention, Imitation/Play, Mastery Motivation, Empathy, Prosocial Peer Relations). In addition, there are three item clusters—Maladaptive, Atypical, and Social Relatedness—which are comprised of relatively rare, but clinically significant problem behaviors (e.g., Tourette's

Syndrome). The reliability and validity of the ITSEA has been examined in several prior studies [34-37].

Child Behavior Checklist (CBCL). The CBCL is a 112-item survey designed to assess socioemotional functioning in children aged 1.5 to 5 years [38]. It was collected as the 18- and 24-month time points. Each item describes a behavior problem (e.g., *acts too young for age*), and caregivers are asked to rate how much the behavior has been a problem for their child in the past 6 weeks (0 = not true; 1 = somewhat/sometimes true; 2 = very/often true). Items are summed to yield two broad factors (Internalizing, Externalizing). The Internalizing factor consists of four subscales (Emotionally Reactive, Anxious/Depressed, Somatic Complaints, Withdrawn). The Externalizing factor consists of two subscales (Attention Problems, Aggressive Behavior). Additionally, there are five DSM scales (Depressive Problems, Anxiety Problems, Autism Spectrum Problems, ADHD Problems, ODD Problems), which were initially developed by having experts from many societies identify problem items that they judged to be very consistent with DSM-IV diagnostic categories [39].

Adult Temperament Questionnaire (ATQ). The short form of the ATQ is a 77-item survey designed to assess general patterns of behavior associated with temperament in adults [40]. Each item is a self-statement (e.g., *I am often late for appointments*) and raters indicate how true each statement is of them using a 7-point Likert scale (1 = *extremely untrue,* 4 = *neither true nor untrue*, 7 = *extremely true*). Each item loads onto one of 13 subscales: Fear, Frustration, Sadness, Discomfort, Activational Control, Attentional Control, Inhibitory Control, Sociability, High-intensity Pleasure, Positive Affect, Neutral Perceptual Sensitivity, Affective Perceptual Sensitivity, Associative Perceptual Sensitivity. Each subscale loads onto one of four factors: Negative Affect, Effortful Control, Extraversion/Surgency, Orienting Sensitivity. This measure was collected at the family's first laboratory visit, which was typically when infants were 4 months of age, but for parents who enrolled later, it was collected at the 8- or 12-month visit.

Eysenck Personality Questionnaire (EPQ). The short version of the EPQ is a 48-item survey designed to assess the core dimensions of personality in adults [41]. Each item takes the form of a question (e.g., *Are you a talkative person?)* and raters indicate their agreement/disagreement by responding with 'Yes' (1) or 'No' (0). Each item loads onto one of three factors which represent the core domains of Eysenck's personality model: Extraversion ("*Do you usually take the initiative in making new friends?*), Neuroticism ("*Do you often feel fed up?*"), and Psychoticism ("*Would you like other people to be afraid of you?*"). Each factor consists of 12 items, and at least 6 responses are needed to compute a scale. This measure was collected at the family's first laboratory visit, which was typically when infants were 4 months of age, but for parents who enrolled later, it was collected at the 8- or 12-month visit.

Cheek & Buss Shyness Scale (CBSS). The CBSS is a 29-item self-report questionnaire assessing shy and social behavior in adults. Each item is a self-statement (e.g., I am socially awkward) and raters circle the number that best indicates how typical the statement is of them (0=not at all characteristic, 1 = *slightly characteristic*, 2 = *moderately characteristic*, 3 = *very characteristic*, 4 = *extremely characteristic*). Each item loads onto one of four subscales (Shyness, Sociability, Fear, Social Anxiety). Shyness [42] is measured from 13 items (e.g., "*I find it hard to talk to strangers*."), Sociability [43] is measured from 5 items (e.g., "*I like to be with people*"), Fear [44] is measured from 5 items (e.g., "*I am easily frightened*."), and Social Anxiety is measured from 6 items (e.g., *It takes me time to overcome my shyness in new situations*). This measure was collected at the family's first laboratory visit, which was typically when infants were 4 months of age, but for parents who enrolled later, it was collected at the 8- or 12-month visit.

Adult Measure of Behavioral Inhibition (AMBI). The AMBI is a 16-item clinical research instrument developed to measure subjective reports of contemporaneous 'trait' inhibition [45]. This instrument is given to respondents 16 years and over as a dimensional quantitative measurement of the temperamental tendency to respond to social novelty and risk stimuli, with inhibition and avoidance. Each item takes the form of a question (e.g., *Do you tend to be chatty in conversation when you are speaking to someone new?)* and raters answer each question using a 3-point scale (0 = *no/hardly ever*; 1 = *some of the time*, 2 = *yes/most of the time*). There are four subscales (Fearful inhibition, Non-approach, Low sociability, Risk Avoidance) that capture specific types of BI. Total scores are generated by summing all items after the negative (for BI) items have been reversed; higher scores indicate a greater degree of inhibition. This measure was collected at the family's first laboratory visit, which was typically when infants were 4 months of age, but for parents who enrolled later, it was collected at the 8- or 12-month visit.

Retrospective Measure of Behavioral Inhibition (RMBI). The RMBI is an 18-item instrument for the retrospective reporting (by adults) of remembered inhibited behaviors in childhood (e.g., during the primary school years ages 5 – 13) [45]. This instrument was designed to capture the principal behavioral indices of "behavioral inhibition to the unfamiliar" as measured and observed in children when assessed in play-laboratory settings. Each item takes the form of a question (e.g., *Were you fearful around other people's pets?*) and raters respond using a 3-point scale (0 = *no/hardly ever*; 1 = *some of the time,* 2 = *yes/most of the time*) by considering how they usually felt, behaved or reacted as a child before the age of 13 (i.e. before high school). 'Do not remember' is an option. Total scores are generated by summing all items after the negative (for BI) items have been reversed; higher scores indicate a greater degree of inhibition. This measure was collected at the family's first laboratory visit, which was typically when infants were 4 months of age, but for parents who enrolled later, it was collected at the 8- or 12-month visit.

Positive and Negative Affect Scales (PANAS). The PANAS is a 20-item survey designed to assess emotionality in adults [46] and was collected at the 4-, 8-, 12-, 18-, and 24-month time points. Each item is a word that depicts a feeling or emotion (e.g., interested, distressed) and raters indicate the extent to which they have felt those emotions in general (1 = *Very slightly/not at all*; 2 = *A little*; 3 = *Moderately*; 4 = *Quite a bit*; 5 = *Extremely*). Values for odd items (positively valanced) are summed to yield a composite for Positive Affect and even items (negatively valanced) are summed to yield a composite for Negative Affect.

Parent Daily Hassles Survey (PDHS-R). The PDHS-R is a 20-item survey designed to assess the frequency and intensity of daily hassles experienced by parents [47]. It was collected at the 4-, 8-, 12-, 18-, and 24-month time points. Each item describes an event that may routinely occur in families with young children (e.g., being nagged, whined at, complained to) and parents note the frequency (rarely, sometimes, a lot, or constantly) and then how much of a 'hassle' the events have been for them within the past 6 months using a 1 to 5 scale. Responses are not child-specific and the survey is not designed to capture relational difficulties with any particular child. The hassles scale can be used in two different ways: totals of frequency and intensity scales, or by deriving 'challenging behavior' and 'parenting tasks' scores from the intensity scale. The challenging behavior total score is obtained by summing seven items from the intensity scale scores and the parenting tasks scale is obtained by summing eight items from the intensity scale.

State-Trait Anxiety Inventory (STAI). The STAI is a 40-item survey designed to assess state and trait anxiety in adults [48]. It was collected at the 4-, 8-, 12-, 18-, and 24-month time points. The STAI is comprised of separate self-report scales for state and trait anxiety. The S-Anxiety scale (STAI Form Y-1) consists of twenty statements that evaluate how respondents feel "right

now, at this moment." The T-Anxiety scale (STAI Form Y-2) consists of twenty statements that assess how people generally feel.

Beck Depression Inventory (BDI). The BDI is a 21-item self-report questionnaire for evaluating the severity of depression in healthy and psychiatric populations [49]. It was collected at the 4-, 8-, 12-, 18-, and 24-month time points. Each item consists of a group of related statements and respondents report how they have been feeling for the past week on a four-point scale (0 = *symptoms absent*, 1 = *mildly*, 2 = *moderately*, 3 = *severe symptoms*). The BDI is scored by adding the highest ratings for all 21 items, for a score range from 0 to 63. Higher scores indicate greater symptom severity. The measure is a reliable and valid assessment of depression with younger [50,51] and older adults [52,53].

Beck Anxiety Inventory (BAI). The BAI is a 21-item self-report questionnaire for evaluating the severity of anxiety in healthy and psychiatric populations [54]. It was collected at the 4-, 8-, 12-, 18-, and 24-month time points. The BAI was specifically designed to distinguish cognitive and somatic symptoms of anxiety from symptoms of depression. Parents rated individual symptoms of anxiety (e.g., fear of losing control) in the past month using a four-point Likert scale (0 = *not at all*, 1 = *mildly*, 2 = *moderately*, 3 = *severely*). The BAI is scored by adding the highest ratings for all 21 items, for a score range from 0 to 63. Higher scores indicate greater symptom severity. Good psychometric properties have been demonstrated for the BAI among multiple outpatient samples [55-57]. Internal consistency of the measure was strong across samples (α = .85 to .92) and adequate test–retest reliability has been demonstrated for anxiety patients (r = .75 to .83 [51,58]. The measure is also moderately correlated with anxiety (r = .36 to .69) and depression measures (r = .25 to .56) completed by psychiatric [54] and normative samples [59].

Confusion, Hubbub, and Order Scale (CHAOS). The CHAOS [60] is a 15-item survey designed to assess 'environmental confusion' (high levels of noise, crowding, traffic pattern) in the home. It was collected at the 4-, 8-, 12-, 18-, and 24-month time points. Each item is a statement (e.g., *There is very little commotion in our home*) with parents responding on a four-point Likert scale (1 = *Very much like your own home*, 2 = *Somewhat like your own home*, 3 = *A little bit like your own home*, 4 = *Not at all like your own home*). A total score is generated by summing all of the items.

Neighborhood Community Survey (ICSPR). The ICSPR is a survey designed to assess adults' perceptions of their neighborhood [61] and was collected at the 4-, 8-, 12-, 18-, and 24-month time points. The survey assesses key neighborhood dimensions, including the dynamic structure of the local community, organizational and political structure, cultural values, informal social control, formal social control, and social cohesion. Variables include measures of the best and worst aspects of living in the region, how long residents had lived in a particular neighborhood, characteristics of their neighborhood, including types of social service agencies available, and if they would consider moving to a different neighborhood and why. Other community variables measure the relationships among neighbors, including how many neighbors a respondent would recognize, how often neighbors socialized, and how often neighbors participated in other activities together. Variables that capture neighborhood social order include respondents' perceptions of neighborhood problems such as litter, graffiti, drinking, drugs, and excessive use of force by police. Respondents were also asked about their normative beliefs regarding violence, money, and various children's behaviors. Victimization variables cover how often the respondent was the victim of a fight with a weapon, a violent argument, a gang fight, sexual assault, robbery, theft, or vandalism. Other variables measure fear of crime and attitudes toward the police. Items can be

aggregated to environmental characteristics, such as social disorder, perceived neighborhood danger, and neighborhood activism.

MacArthur Bates Communicative Development Inventory (MB-CDI). The MB-CDI is a screening measure of early communicative development for children under 30 months and was collected at the 24-month assessment. The instrument captures young children's developing language abilities, including vocabulary comprehension, production, gestures, and grammar. For toddlers, there are two equivalent checklists, each containing 100 items (52-53% nouns, 17-18% verbs, 15-17% adjectives/adverbs, 13-15% pronouns). For each item, parents indicate (Yes or No) if their child understands that word. The total number of words understood and produced is calculated via summing. When derived from the full CDI for the same children, the two short forms correlated with each other at r = .99 [28].

Geocoding

Geocoding based on demographics data was implemented to provide an additional measure of the environment. The two-step process consists of obtaining the address of the participant and deriving the census tract and block group for that address, or census geography types associated with the address. After collecting the census tract and block group, the census geography types were entered into americanfactfinder.com, which provides sociodemographic variables for that specific geographic location indicating variation in factors such as socioeconomic status, household structure, and access to transportation. The following variables were measured: highest educational attainment of the general population, highest educational attainment of the female population, the school dropout rate for the population age 16 to 19 years, employment statuses, labor force for the population 16 years and older, poverty status of families, household income, and households with earnings. Household information was derived from two variables household size and household type. Access to transportation to work were measured through means of transportation to work and travel time to work.

Telomere Length Assays

Telomere length was derived from buccal cells collected from both 24-month-olds and their primary caregivers using Isohelix swab kits, according to standardized swabbing procedures (Cell Projects Ltd., Kent, UK). Samples collected prior to the COVID-19 pandemic were collected in the lab and were immediately placed in a -80 Celsius freezer. Samples collected during and after the COVID-19 pandemic were collected at home while experimenters supervised via Zoom to ensure the swab protocol was followed. These samples were immediately placed in a -20 Celsius freezer (standard home freezer temperature) in the participant home for short-term storage, and later picked up by an experimenter at an arranged time and transferred to a -80 Celsius freezer for long-term storage. All samples collected from across sites were shipped to Penn State using dry ice to maintain optimal temperature (-20 to -80 Celsius) to avoid the freeze-thaw cycle, a process which has been shown to impact telomere length assays [62]. Any deviations from the protocol were noted, and samples were excluded accordingly from analyses. We obtained two samples each from child and parent, and telomere length for each individual was averaged from their two samples. DNA will be extracted, and telomere length assays will be conducted by trained specialists, using a protocol adapted from O'Callaghan et al. [63].

Overarching analytic approach

As noted, LAnTs had two overarching questions of interest**.** First, we will test the integral bias, moderation, and acquisition models outlined by Field and Lester [64]. Second, we will examine the extent to which the gradient of individual attention growth curves predicts behavioral inhibition at age two.

Question 1 will be tested through a hierarchical growth model [65] in which observations (level 1) over time are nested within children (level 2) nested within sites (level 3). First, the best fitting growth trajectory for attentional biases over time will be established by comparing the following first-order fractional polynomials: inverse cube, inverse square, inverse, inverse square root, log, square root, linear, square, and cube [66]. The trajectory with the smallest AIC will be retained in the model. Variance in baseline levels of attentional bias will be modeled by fitting a random effect of the intercept of the growth curve, whereas individual differences in the trajectory of attentional bias over time will be modeled by allowing the slope of the growth curve to vary across children. To examine the role of temperament, negative affect will be added as a time-covarying fixed effect, and the interaction between time and negative affect will establish how the growth curve is moderated by negative affect. The integral bias model predicts a zero gradient for the growth curve, no significant interaction between the gradient of the curve and negative affect, and significant variance in intercepts (baseline attentional bias). The moderation and acquisition models both predict relatively low variance in intercepts, a non-zero gradient for the growth curve, and a significant interaction between negative affect and the gradient of the growth curve. These models can be distinguished in that the moderation model predicts increasingly negative gradients as affect changes whereas the acquisition model predicts increasingly positive gradients as affect changes. Other potential factors will similarly be tested by extending the model. For example, biological markers will be added to the model as time-varying fixed effects. The hypothesis is, in essence, tested by looking at the interaction between these markers and the (assumed) time by negative affect interaction in the basic model.

To examine question 2, predictors that improve the fit of the model will be retained such that the endpoint of testing question 1 is a parsimonious model of the developmental trajectory of attention to threat and the factors that moderate it. This model will be used to test question 2 by using gradients of the trajectory of attention bias, and moderators of those gradients, as predictors of BI at age two. If the integral bias model is supported, the intercepts, rather than gradients of attention to threat, will be used. Exploratory analyses will incorporate sex in the analyses, testing previous findings suggesting that boys show higher levels of BI and are at increased risk for poor outcomes [19,67-69].

References

1. Vallorani A, Fu X, Morales S, LoBue V, Buss KA, Pérez-Edgar K. Variable- and Person-Centered Approaches to Affect-Biased Attention in Infancy Reveal Unique Relations with Infant Negative Affect and Maternal Anxiety. *Scientific Reports.* 2021;11.
2. MacLeod C, Mathews A, Tata P. Attentional bias in emotional disorders. *Journal of Abnormal Psychology.* 1986;95:15-20.
3. Tottenham N, Tanaka J, Leon A, et al. The NimStim set of facial expressions: Judgments from untrained research participants. *Psychiatry Research.* 2009;168:242-249.
4. Pérez-Edgar K, Morales S, LoBue V, et al. The impact of negative affect on attention patterns to threat across the first two years of life. *Developmental Psychology.* 2017;53:2219-2232.
5. Morales S, Brown KM, Taber-Thomas BC, LoBue V, Buss KA, Pérez-Edgar K. Maternal anxiety predicts attentional bias towards threat in infancy. *Emotion.* 2017;17:874-883.
6. Peltola MJ, Leppanen JM, Palokangas T, Hietanen JK. Fearful faces modulate looking duration and attention disengagement in 7-month-old infants. *Developmental Science.* 2008;11:60-68.
7. Canfield RL, Haith MM. Young infants' visual expectations for symmetric and asymmetric stimulus sequences. *Developmental Psychology.* 1991;27(2):198.
8. Fu X, Morales S, LoBue V, Buss KA, Pérez-Edgar K. Temperament moderates developmental changes in vigilance to emotional faces in infants: Evidence from an eye-tracking study. *Developmental Psychobiology.* 2020;62:339-352.
9. Fu X, Taber-Thomas BC, Pérez-Edgar K. Frontolimbic functioning during threat-related attention: Relations to early behavioral inhibition and anxiety in children. *Biological Psychology.* 2017;122:98-109.
10. Morales S, Pérez-Edgar K, Buss KA. Attention biases towards and away from threat mark the relation between early dysregulated fear and the later emergence of social withdrawal. *Journal of Abnormal Child Psychology.* 2015;43:1067-1078.
11. Thai N, Taber-Thomas BC, Pérez-Edgar K. Neural correlates of attention biases, behavioral inhibition, and anxiety in children: An ERP study. *Developmental Cognitive Neuroscience.* 2016;19:200-210.
12. Ohman A, Flykt A, Esteves F. Emotion drives attention: Detecting the snake in the grass. *Journal of Experimental Psychology: General.* 2001;130:466-478.
13. Fox NA, Henderson HA, Rubin KH, Calkins SD, Schmidt LA. Continuity and discontinuity of behavioral inhibition and exuberance: Psychophysiological and behavioral influences across the first four years of life. *Child Development.* 2001;72:1-21.
14. Kagan J, Snidman N. Infant predictors of inhibited and uninhibited profiles. *Psychological Science.* 1991;2:40-44.
15. Goldsmith HH, Rothbart MK. *The laboratory temperament assessment battery (LAB-TAB).* Madison: University of Wisconsin Press; 1993.
16. García Coll C, Kagan J, Reznick JS. Behavioral inhibition in young children. *Child Development.* 1984;55:1005-1019.
17. Buss KA, Davis EL, Ram N, Coccia M. Dysregulated fear, social inhibition, and respiratory sinus arrhythmia: A replication and extension. *Child Development.* 2018;89(3):e214-e228.

18. Dollar JM, Stifter CA, Buss KA. Exuberant and inhibited children: Person-centered profiles and links to social adjustment. *Developmental Psychology.* 2017;53(7):1222.
19. Fox NA, Snidman N, Haas SA, Degnan KA, Kagan J. The relations between reactivity at 4 months and behavioral inhibition in the second year: Replication across three independent samples. *Infancy.* 2015;20(1):98-114.
20. Berntson GG, Quigley KS, Jang JF, Boysen ST. An approach to artifact identification: application to heart period data. *Psychophysiology.* 1990;27:586-598.
21. Degnan KA, Hane AA, Henderson HA, Moas OL, Reeb-Sutherland BC, Fox NA. Longitudinal stability of temperamental exuberance and social-emotional outcomes in early childhood. *Developmental Psychology.* 2011;47:765-780.
22. Luck SJ. *An introduction to the event-related potential technique.* MIT press; 2014.
23. Anaya B, Vallorani A, Pérez-Edgar K. Individual dynamics of delta-beta synchrony: Using a multilevel framework to examine trait- and state-level differences in relation to Behavioral Inhibition and social anxiety. *Journal of Child Psychology & Psychiatry.* 2021.
24. Phelps RA, Brooker RJ, Buss KA. Toddlers' dysregulated fear predicts delta–beta coupling during preschool. *Developmental Cognitive Neuroscience.* 2016;17:28–34.
25. Herwig U, Satrapi P, Schönfeldt-Lecuona C. Using the international 10-20 EEG system for positioning of transcranial magnetic stimulation. *Brain Topography.* 2003;16(2):95-99.
26. Hane AA, Fox NA, Henderson HA, Marshall PJ. Behavioral reactivity and approach-withdrawal bias in infancy. *Developmental Psychology.* 2008;44:1491-1496.
27. Donoghue T, Haller M, Peterson EJ, et al. Parameterizing neural power spectra into periodic and aperiodic components. *Nature neuroscience.* 2020;23(12):1655-1665.
28. Fenson L. *MacArthur-Bates communicative development inventories.* Paul H. Brookes Publishing Company Baltimore, MD; 2007.
29. Putnam SP, Helbig AL, Gartstein MA, Rothbart MK, Leerkes E. Development and assessment of short and very short forms of the infant behavior Questionnaire-Revised. *Journal of Personality Assessment.* 2014;96:445-458.
30. Parade SH, Leerkes EM. The reliability and validity of the Infant Behavior Questionnaire-Revised. *Infant Behavior and Development.* 2008;31(4):637-646.
31. Goldsmith HH, Campos JJ. The structure of temperamental fear and pleasure in infants: A psychometric perspective. *Child Development.* 1990;61(6):1944-1964.
32. Gartstein MA, Marmion J. Fear and positive affectivity in infancy: Convergence/discrepancy between parent-report and laboratory-based indicators. *Infant Behavior and Development.* 2008;31(2):227-238.
33. Goldsmith HH. Studying temperament via construction of the Toddler Behavior Assessment Questionnaire. *Child development.* 1996;67(1):218-235.
34. Carter AS, Briggs-Gowan MJ, Jones SM, Little TD. The Infant–Toddler Social and Emotional Assessment (ITSEA): Factor structure, reliability, and validity. *Journal of Abnormal Child Psychology.* 2003;31:495-514.
35. Briggs-Gowan MJ, Carter AS. Preliminary acceptability and psychometrics of the infant–toddler social and emotional assessment (ITSEA): A new adult-report questionnaire. *Infant Mental Health Journal: Official Publication of The World Association for Infant Mental Health.* 1998;19(4):422-445.
36. Briggs-Gowan MJ, Carter AS, Bosson-Heenan J, Guyer AE, Horwitz SM. Are infant-toddler social-emotional and behavioral problems transient? *Journal of the American Academy of Child & Adolescent Psychiatry.* 2006;45(7):849-858.

37. Briggs-Gowan MJ, Carter AS. *BITSEA: Brief infant-toddler social and emotional assessment. Examiner's manual.* Harcourt Assessment; 2006.
38. Achenbach TM, Edelbrock C. *Manual for the Child Behavior Checklist and revised child behavior profile.* Burlington, VT: University of Vermont, Department of Psychiatry; 1983.
39. Achenbach TM, Rescorla LA. The Achenbach System of Empirically Based Assessment (ASEBA): Applications in forensic contexts. 2013.
40. Evans DE, Rothbart MK. Development of a model for adult temperament. *Journal of Research in Personality.* 2007;41:868-888.
41. Eysenck H. Manual of the Eysenck Personality Questionnaire (Junior & Adult). *San Diego: Edits/Educational and Industrial Testing Service.* 1975.
42. Cheek JM. The revised Cheek and Buss shyness scale. *Unpublished manuscript, Wellesley College, Wellesley, MA.* 1983;2181.
43. Cheek J, Buss A. Shyness and sociability. *Journal of Personality and Social Psychology.* 1981;41:330-339.
44. Buss AH, Plomin R. *A temperament theory of personality development.* Wiley-Interscience; 1975.
45. Gladstone GL, Parker GB. Measuring a behaviorally inhibited temperament style: Development and initial validation of new self-report measures. *Psychiatry Research.* 2005;135:133-143.
46. Watson D, Clark LA, Tellegen A. Development and validation of brief measures of positive and negative affect: the PANAS scales. *Journal of personality and social psychology.* 1988;54(6):1063.
47. Crnic KA, Booth CL. Mothers' and fathers' perceptions of daily hassles of parenting across early childhood. *Journal of Marriage and the Family.* 1991;53(4):1043–1050.
48. Spielberger C. *Manual for the State-Trait Anxiety Inventory (STAI).* PaloAlto, CA: Consulting Psychologists Press; 1983.
49. Beck A, Ward C, Mendelson M, Mock J, Erbaugh J. An inventory for measuring depression. *Archives of General Psychiatry.* 1961;4:561-571.
50. Beck AT, Steer RA. Internal consistencies of the original and revised Beck Depression Inventory. *Journal of clinical psychology.* 1984;40(6):1365-1367.
51. Beck A, Steer R, Garbin M. Psychometric properties of the Beck Depression Inventory: Twenty-five years of evaluation. *Clinical Psychology Review.* 1988;8:77-100.
52. Stanley MA, Novy DM, Bourland SL, Beck JG, Averill PM. Assessing older adults with generalized anxiety: a replication and extension. *Behaviour Research and Therapy.* 2001;39(2):221-235.
53. Gallagher D, Nies G, Thompson LW. Reliability of the Beck Depression Inventory with older adults. *Journal of consulting and clinical psychology.* 1982;50(1):152.
54. Beck A, Epstein N, Brown G, Steer R. An inventory for measuring clinical anxiety: Psychometric properties. *Journal of Consulting and Clinical Psychology.* 1988;56:893-897.
55. Morin CM, Landreville P, Colecchi C, McDonald K, Stone J, Ling W. The Beck Anxiety Inventory: psychometric properties with older adults. *Journal of Clinical Geropsychology.* 1999;5(1):19-29.
56. Steer RA, Willman M, Kay PA, Beck AT. Differentiating elderly medical and psychiatric outpatients with the Beck Anxiety Inventory. *Assessment.* 1994;1(4):345-351.

57. Wetherell JL, Areán PA. Psychometric evaluation of the Beck Anxiety Inventory with older medical patients. *Psychological Assessment.* 1997;9(2):136.
58. de Beurs E, Wilson KA, Chambless DL, Goldstein AJ, Feske U. Convergent and divergent validity of the Beck Anxiety Inventory for patients with panic disorder and agoraphobia. *Depression and anxiety.* 1997;6(4):140-146.
59. Osman A, Kopper BA, Barrios FX, Osman JR, Wade T. The Beck Anxiety Inventory: Reexamination of factor structure and psychometric properties. *Journal of clinical psychology.* 1997;53(1):7-14.
60. Matheny AP, Washs TD, Ludwig JL, Philips K. Bringing order out of chaos: Psychometric characteristics of the confusion, hubbub, and order scale. *Journal of Applied Developmental Psychology.* 1995;16:429-444.
61. Earls FJ, Brooks-Gunn J, Raudenbush SW, Sampson RJ. *Project on Human Development in Chicago Neighborhoods: Community Survey, 1994-1995.* Ann Arbor, MI: Inter-university Consortium for Political and Social Research; 2007.
62. Lin J, Smith DL, Esteves K, Drury S. Telomere length measurement by qPCR–Summary of critical factors and recommendations for assay design. *Psychoneuroendocrinology.* 2019;99:271-278.
63. O'Callaghan NJ, Dhillon VS, Thomas P, Fenech M. A quantitative real-time PCR method for absolute telomere length. *Biotechniques.* 2008;44(6):807-809.
64. Field AP, Lester KJ. Is there room for ‘development’ in developmental models of information processing biases to threat in children and adolescents? *Clinical Child and Family Psychology Review.* 2010;13:315-332.
65. Singer JD, Willett JB. *Applied Longitudinal Data Analysis: Modeling Change and Event Occurrence.* Oxford: OUP; 2003.
66. Long JD. *Longitudinal Data Analysis for the Behavioral Sciences Using R.* Thousand Oaks, CA: Sage; 2012.
67. Else-Quest NM, Hyde JS, Goldsmith HH, Van Hulle CA. Gender differences in temperament: A meta-analysis. *Psychological bulletin.* 2006;132(1):33.
68. Henderson HA, Fox NA, Rubin KH. Temperamental contributions to social behavior: The moderating roles of frontal EEG asymmetry and gender. *Journal of the American Academy of Child and Adolescent Psychiatry.* 2001;40:68-74.
69. Howarth GZ, Guyer AE, Pérez-Edgar K. Young children’s affective responses to evaluative feedback from peers: A computer-based task sensitive to variation in temperamental shyness and gender. *Social Development.* 2013;22:146-162.
